# Generative artificial intelligence (GAI) usage guidelines for scholarly publishing: a cross-sectional study of medical journals

**DOI:** 10.1186/s12916-025-03899-1

**Published:** 2025-02-11

**Authors:** Shuhui Yin, Simu Huang, Peng Xue, Zhuoran Xu, Zi Lian, Chenfei Ye, Siyuan Ma, Mingxuan Liu, Yuanjia Hu, Peiyi Lu, Chihua Li

**Affiliations:** 1 Applied Linguistics & Technology, Department of English, Iowa State University, Ames, IA USA; 2 Center for Data Science, Zhejiang University, Hangzhou, Zhejiang China; 3 Institute of Chinese Medical Sciences, University of Macau, Zhuhai, Macao SAR, China; 4 Centre for Pharmaceutical Regulatory Sciences, University of Macau, Zhuhai, Macao SAR, China; 5 Faculty of Health Sciences, University of Macau, Zhuhai, Macao SAR, China; 6 Graduate Group in Genomics and Computational Biology, Perelman School of Medicine, University of Pennsylvania, Philadelphia, PA USA; 7 Center for Health Equity & Urban Science Education, Teachers College, Columbia University, New York, NY USA; 8 International Research Institute for Artificial Intelligence, Harbin Institute of Technology, Shenzhen, Guangdong China; 9 Department of Communication, University of Macau, Zhuhai, Macao SAR, China; 10 Department of Social Work and Social Administration, University of Hong Kong, Hong Kong SAR, China; 11 Survey Research Center, Institute for Social Research, University of Michigan, Ann Arbor, MI USA

**Keywords:** GAI usage guidelines, Scholarly publishing, Medical journals, Coverage of recommendations, Type of recommendations, SJR score

## Abstract

**Background:**

Generative artificial intelligence (GAI) has developed rapidly and been increasingly used in scholarly publishing, so it is urgent to examine guidelines for its usage. This cross-sectional study aims to examine the coverage and type of recommendations of GAI usage guidelines among medical journals and how these factors relate to journal characteristics.

**Methods:**

From the SCImago Journal Rank (SJR) list for medicine in 2022, we generated two groups of journals: top SJR ranked journals (*N* = 200) and random sample of non-top SJR ranked journals (*N* = 140). For each group, we examined the coverage of author and reviewer guidelines across four categories: no guidelines, external guidelines only, own guidelines only, and own and external guidelines. We then calculated the number of recommendations by counting the number of usage recommendations for author and reviewer guidelines separately. Regression models examined the relationship of journal characteristics with the coverage and type of recommendations of GAI usage guidelines.

**Results:**

A higher proportion of top SJR ranked journals provided author guidelines compared to the random sample of non-top SJR ranked journals (95.0% vs. 86.7%, *P* < 0.01). The two groups of journals had the same median of 5 on a scale of 0 to 7 for author guidelines and a median of 1 on a scale of 0 to 2 for reviewer guidelines. However, both groups had lower percentages of journals providing recommendations for data analysis and interpretation, with the random sample of non-top SJR ranked journals having a significantly lower percentage (32.5% vs. 16.7%, *P* < 0.05). A higher SJR score was positively associated with providing GAI usage guidelines for both authors (all *P* < 0.01) and reviewers (all *P* < 0.01) among the random sample of non-top SJR ranked journals.

**Conclusions:**

Although most medical journals provided their own GAI usage guidelines or referenced external guidelines, some recommendations remained unspecified (e.g., whether AI can be used for data analysis and interpretation). Additionally, journals with lower SJR scores were less likely to provide guidelines, indicating a potential gap that warrants attention. Collaborative efforts are needed to develop specific recommendations that better guide authors and reviewers.

**Supplementary Information:**

The online version contains supplementary material available at 10.1186/s12916-025-03899-1.

## Background

ChatGPT, a chatbot powered by generative artificial intelligence (GAI) through large language models, was released in November 2022. It can generate responses based on statistical language patterns and is easily accessible to people without technical expertise [1]. Its multifaceted capabilities and potential applications within diverse contexts have attracted over 100 million active users and billions of visits [2–4]. Consequently, many researchers have been actively exploring the potential applications of ChatGPT and similar tools such as Bing AI, Gemini, and Claude in scholarly publishing, including manuscript preparation and peer review [5–9]. To date, hundreds and thousands of medical and scientific publications have reported and discussed the use and impact of GAI tools in scholarly publishing [10–15].

Specifically, during manuscript preparation, researchers have been found using GAI tools to conduct literature review, formulate study design, analyze data, interpret results, and generate scientific reports [16–18]. A recent large-scale study based on 950,965 papers published in 2020–2024 found a major increase in the use of GAI for writing these papers after the release of ChatGPT [19]. The increase ranges from over 6% to almost 20% across different disciplines. During peer review, GAI has been used to summarize manuscript contents, review code, check methods, and even draft comments and feedback [10, 20, 21]. A study based on peer reviews to AI conferences reported between 6.5% and 16.9% of related text submitted as peer reviews could have been substantially modified by GAI tools [22]. These findings highlight that such tools have and will continue to transform scholarly publishing.

However, GAI is far from perfect and can lead to multiple concerns [23–25]. Major concerns regarding its role in scholarly publishing include the eligibility of these tools for authorship, the risk of producing misleading or inaccurate information, breaches of data privacy and confidentiality, and undisclosed and illicit usage, as well as challenges to integrity and originality [26–29]. In response, many journals, organizations, and publishers have started to provide GAI usage guidelines for authors and reviewers and made continuous updates [30–32]. These guidelines serve as an important communication tool to guide authors and reviewers on topics such as authorship criteria and ethical standards [33–35]. For example, medical publication organizations, and publishers, such as the International Committee of Medical Journal Editors (ICMJE) and Elsevier, have decided that GAI should not be listed as authors because they cannot be accountable for the submitted work [36, 37]; some journals require authors to document details of GAI usage and fact-check GAI-generated contents [38]; and some journals strictly prohibit the use of GAI in peer review [39]. These developments show the new challenges posed by GAI and the scientific community’s corresponding adaptations.

Despite a fast-growing increase in discussions on GAI usage in scientific research, few have examined GAI usage guidelines systematically. For example, one study examined GAI usage guidelines for authors based on the 100 largest publishers and top 100 highly ranked journals of different disciplines, and it found less than a quarter of these publishers and around 85% of the top journals provided author guidelines [40]. The other study examined author disclosure recommendations for GAI usage in 125 nursing journals and found less than 40% of them had explicit instructions [41]. They offered valuable insights into the evolving ethical standards and practices of this rapidly changing field.

However, many other important aspects of GAI usage guidelines remain less explored or unknown. First, few or no existing studies examined GAI guidelines for reviewers, ignoring the equal importance of peer review in scholarly publishing. Second, previous studies either focused on top ranked journals or exclusively on nursing journals [40, 41], leading to an incomplete assessment of such guidelines across non-top ranked medical journals and a lack of empirical comparisons with top ranked journals. Third, little exploration has been made on the relationship between journal characteristics and GAI guidelines for authors and reviewers. To address these gaps, our study focused on medical journals and included both top and non-top SJR ranked journals. We aimed to delineate the coverage and types of recommendations of GAI guidelines for authors and reviewers as well as external guidelines referred by these journals. We also examined potential relationships between journal characteristics and the coverage and number of recommendations of different GAI guidelines. By coverage, we refer to whether the journal provides guidelines for authors and reviewers. By type of recommendations, we refer to the recommendations that the GAI usage guidelines provide regarding various aspects of academic practices in scholarly publishing. These together would provide a thorough overview of current practices and contribute to the ongoing development of GAI usage guidelines across medical journals.

## Methods

### Journal selection

We followed the STROBE guidelines in conducting and reporting this study (Additional file 1: Supplementary Table 1). Based on the SCImago Journal Rank (SJR), two groups of journals were selected from the journal ranking list for medicine in 2022: top SJR ranked journals and a random sample of non-top SJR ranked journals [42]. Top SJR ranked journals refer to the first 200 journals ranked based on SJR scores. The random sample of non-top SJR ranked journals refers to journals covering the entire range of SJR scores, excluding the first 200 SJR ranked journals. The detailed selection process is outlined below.

**Fig. 1:**
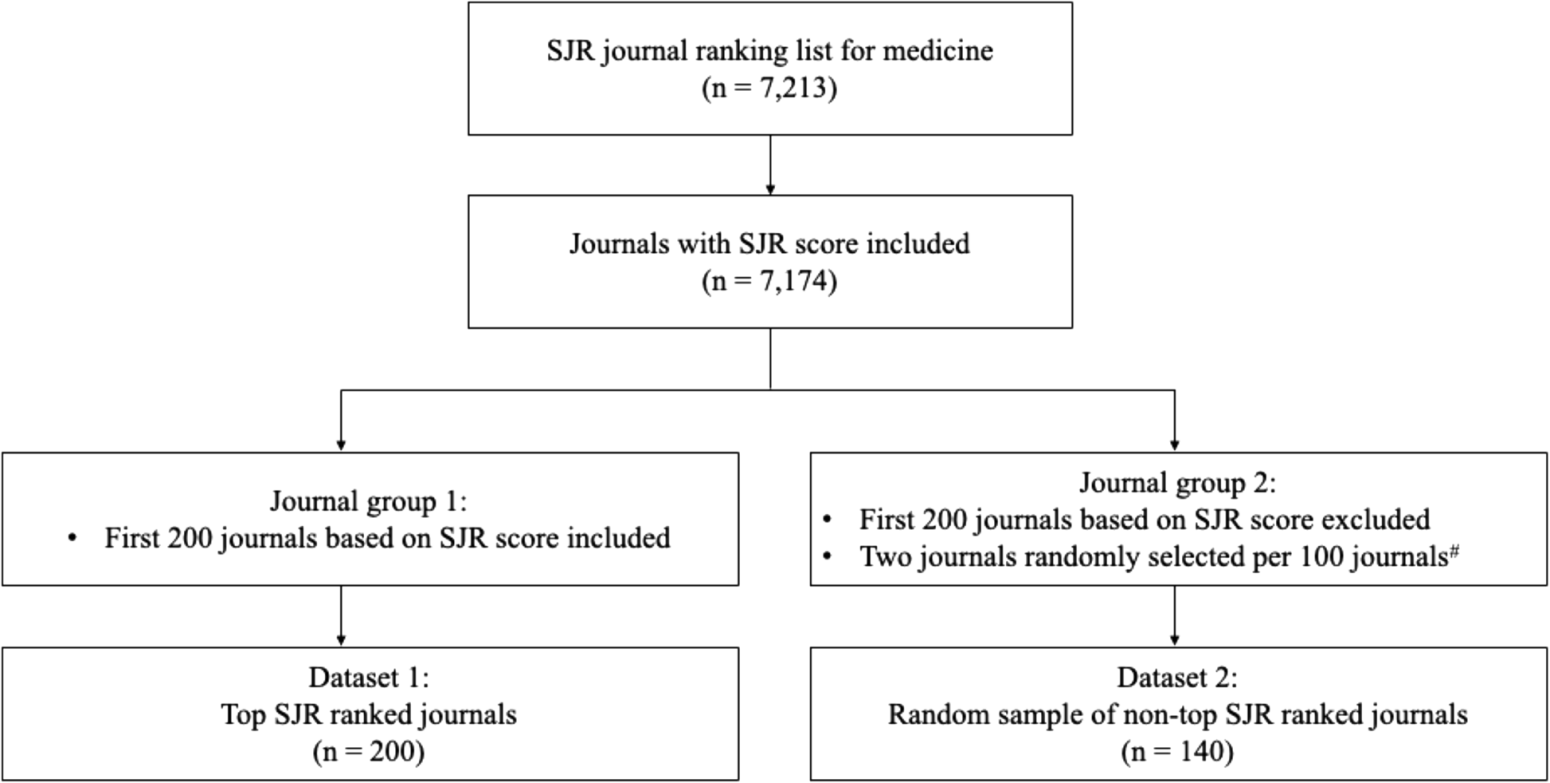
Flowchart of journal selection. ^#^ A stratified random sampling method was implemented

A total of 7213 medical journals were initially extracted from the 2022 SJR database (Fig. 1). Each journal was ranked in descending order according to its 2022 SJR score, a measure of the number of citations received by its articles that contextualizes the journal’s prestige and popularity within the academic community. Journals without SJR scores were excluded, leaving 7174 journals. The exclusion was made for two reasons. First, journals with SJR scores can be ranked for subsequent analysis. Second, the 39 journals without SJR scores only accounted for less than 1% of the total medical journals in the database. Nonetheless, a sensitivity analysis was performed by randomly selecting one journal from the 39 journals without SJR scores and assigning it to a lower-tier category to determine if the exclusion would affect the results.

The group of top SJR ranked journals was selected based on two reasons. First, high-ranking journals have been commonly used in prior research, enabling us to compare our findings with previous studies [40]. Second, top SJR ranked journals are typically regarded as representing the highest standard and often provide extensive discussions on the use of GAI or relevant tools. These journals will provide rich information on the current state of GAI usage guidelines [7, 43]. To obtain a group of top SJR ranked journals, referring to sample size in previous studies (ranging from 100 to 200) [40], we selected the first 200 ranked journals from the 2022 SJR database. These top SJR ranked journals represent the top 2.8% of all journals.

To provide a comparative view of GAI usage guidelines in non-top ranked medical journals which had more diverse background and relatively limited academic resources, we also generated a group of random sample of non-top SJR ranked journals. This inclusion allowed us to compare GAI usage guidelines between top SJR ranked journals and non-top SJR ranked journals. We excluded the first 200 journals because they had been sampled in the top SJR ranked journals. We employed a stratified random sampling method [44]. From each stratum of 100 ranked journals, two journals were randomly selected using a random number generator, resulting in a total of 140 journals. This was done to ensure that we captured the entire range of journals and achieved a balanced random sampling from each stratum. Non-English journals were excluded.

### Data collection and extraction

For each journal selected above, at least two members of our research team independently carried out a thorough search for the guidelines, which were publicly available on each journal’s website. This was to identify any content related to the GAI usage. The two researchers carefully piloted the screening process and achieved a high level of agreement, with over 95% of the content independently extracted being identical. The following information was extracted to create two datasets for top SJR ranked journals and random sample of non-top SJR ranked journals respectively: journal characteristics and three types of GAI usage guidelines (Table 1). Journal characteristics included five items: journal name, publisher, SJR score, region, and focus area. The focus area was classified into two groups: general medicine and specialty areas [45]. Three types of GAI usage guidelines included author guidelines, reviewer guidelines, and references to external guidelines. The data collection took place on July 24–30, 2024. This 7-day window was selected to balance the need for up-to-date information with the requirement to minimize significant changes in guidelines, as expected in a cross-sectional study.

**Table 1:** Journal characteristics and three types of generative artificial intelligence (GAI) usage guidelines

**Items**	**Journal characteristics**	**Three types of GAI usage guidelines**		
		**Author guidelines** **(Y/N)**	**Reviewer guidelines** **(Y/N)**	**References to external guidelines** **(Y/N)**
**1**	Journal name	Usage permission	Usage permission	COPE
**2**	Publisher	Language editing	Language editing	ICMJE
**3**	SJR score	Manuscript writing	Usage documentation	WAME
**4**	Region	Data analysis and interpretation		
**5**	Focus area	Image generating		
**6**		Fact-checking		
**7**		Usage documentation		
**8**		Authorship eligibility		

Footnote: Y, Yes; N, No; COPE, Committee on Publication Ethics; ICMJE, International Committee of Medical Journal Editors; WAME, World Association of Medical Editors

The coverage of GAI usage guidelines was classified into four categories: no guidelines, external guidelines only, own guidelines only, and own and external guidelines. Following the previous study, we define “own” guidelines to be both the journal and publisher level [40]. For journal-level guidelines, we refer to instances where journals provide GAI usage guidelines on their official websites. However, if a journal did not provide GAI usage guidelines on its official websites, we used the publisher’s guidelines as a proxy, but only when the journal’s author guidelines, reviewer guidelines, or ethics page explicitly recommended or linked directly to the publisher’s guidelines. Many journals referred to external guidelines formulated by publishing organizations for authors and reviewers. For these external guidelines on GAI usage, we focused on standards formulated by three groups: the Committee on Publication Ethics (COPE), ICMJE, and the World Association of Medical Editors (WAME). If a journal did not provide own guidelines but referenced external guidelines, it was classified as “external guidelines only.” If the journal neither provided own guidelines nor referenced external guidelines, it was classified as “no guidelines.” Conversely, if the journal provided own guidelines and referenced external guidelines, it was classified as “own and external guidelines.”

A detailed examination of available guidelines was conducted to identify a comprehensive list of available GAI usage recommendations [46]. First, two researchers of our team independently reviewed all extracted GAI usage guidelines and coded the content inductively. We documented the codes and met to discuss the initial coding. During this process, we found a high level of consistency in our coding and resolved any minor discrepancies through discussion. Second, we referred to items identified by pioneering studies in this field to complement the above coding [25, 40, 41, 47]. As a result, we developed a checklist consisting of eight items for author guidelines and three items for reviewer guidelines. These items represent both the standard components of the medical research process and critical aspects widely discussed in the literature.

For author guidelines, the eight items include one about usage permission and seven about the type of recommendations on GAI usage: language editing, manuscript writing, data analysis and interpretation, image generating, fact-checking, usage documentation, and authorship eligibility. Usage permission refers to whether GAI tools are permitted to be used in manuscript preparation. Language editing, manuscript writing, data analysis and interpretation, and image generating refer to whether GAI tools are permitted to be used in each of these manuscript development steps. Fact-checking refers to whether authors are required to check and verify AI-generated content. Usage documentation refers to whether authors are required to disclose and document the use of GAI tools. Authorship eligibility refers to whether AI owns authorship and is permitted to be listed as an author.

Similarly, for reviewer guidelines, we identified three items including one about usage permission and two types of recommendations: language editing and usage documentation. We further coded each item as “Yes” or “No” according to the above definition. The Cohen’s kappa coefficients ranged from 0.85 to 0.91 for the seven types of recommendations, suggesting high inter-coder reliability [48].

Notably, for usage permission, journals typically provided explicit instructions on whether GAI usage was permitted for authors or reviewers. While we further coded the seven types of recommendations separately, journals permitting GAI usage generally permit it for at least one of the seven recommended types.

For journals permitting GAI usage, we further analyzed their number of recommendations in author guidelines and reviewer guidelines separately. The author guidelines account for seven recommendations of GAI usage and the number of recommendations range from 0 to 7; the reviewer guidelines account for two recommendations of GAI usage and the number of recommendations range from 0 to 2. These numbers were derived from the above coding: a value of “1” was assigned for either “Yes” or “No” to each type of recommendation; and a value of “0” if the practice was not specified. A higher number of recommendations indicates that more types were addressed in the journals permitting GAI usage.

Additionally, we coded and calculated the number of recommendations for each of the three external guidelines to examine their instructions for GAI usage and align them with our number of recommendations for journals.

### Statistical analysis

We summarized and compared the characteristics and coverage of different usage guidelines between top SJR ranked journals and random sample of non-top SJR ranked journals. Additionally, we examined the relationship of journal characteristics with the coverage and type of recommendations of GAI usage guidelines after pooling the two groups of journals. Statistical differences between the two groups were using appropriate tests based on the data type. For categorical variables, we used Chi-Square tests. For continuous variables, we used *t*-tests. Fisher’s exact test was used when the count in certain cells was less than five. We used multinominal logistic regression to examine relationships between journal characteristics and the coverage of author or reviewer guidelines, since there was no gold standard for these different coverage categories. We used linear regression to relationships between journal characteristics and the number of recommendations of these guidelines. All analyses were adjusted for the sampling weights. We conducted all analyses using R 4.2. All data used for this study was provided in Additional file 1: Supplementary Tables 2, 3, and 4. All our data and code used for data analysis, along with relevant statistical outputs, have been deposited in the GitHub repository: https://github.com/simuh34/GAI-usage-guidelines-across-medical-journals [49].

## Results

Table 2 summarizes journal characteristics and the coverage of different GAI usage guidelines. The 200 top journals had a median SJR score of 4.7, and 198 of them (99.0%) were based in either Northern America or Western Europe. The 140 random sample of non-top SJR ranked journals had a median SJR score of 0.5, and they were more evenly distributed across the three regions. The two groups of journals have a similar distribution in terms of focus area, with approximately one fifth of the journals focusing on general medicine and around four-fifths on specialty areas. For author guidelines, 95.0% of the top SJR ranked journals and 86.7% of the random sample of non-top SJR ranked journals (*P* < 0.01) provided any author guidelines including external guidelines only, own guidelines only, and own and external guidelines. For reviewer guidelines, 92.5% of the top SJR ranked journals and 86.7% of the random sample of non-top SJR ranked journals (*P* = 0.08) provided any of the reviewer guidelines including external guidelines only, own guidelines only, and own and external guidelines.

**Table 2:** Journal characteristics and coverage of different GAI usage guidelines

	**Top SJR ranked journals** (*n* = 200)	Random sample of non-top SJR ranked journals (*n* = 140)	*P* value^#^
**Journal characteristics**			
SJR score, median (Q1, Q3)	4.7 (3.6, 7.3)	0.5 (0.2, 0.9)	< 0.01
Region, n (%)			
Northern America	96 (48.0)	37 (26.5)	< 0.01
Western Europe	102 (51.0)	50 (35.5)	
Other regions^*^	2 (1.0)	53 (38.0)	
Focus area, n (%)			
General medicine	40 (20.0)	31 (22.0)	0.65
Specialty areas	160 (80.0)	109 (78.0)	
**GAI guidelines**^¶^			
Author guidelines, n (%)			
No guidelines	10 (5.0)	19 (13.3)	< 0.01
External guidelines only	30 (15.0)	49 (35.1)	
Own guidelines only	8 (4.0)	5 (3.6)	
Own & external guidelines	152 (76.0)	67 (48.0)	
Reviewer guidelines, n (%)			
No guidelines	15 (7.5)	19 (13.3)	< 0.01
External guidelines only	87 (43.5)	86 (61.7)	
Own guidelines only	3 (1.5)	5 (3.6)	
Own & external guidelines	95 (47.5)	30 (21.5)	

Footnote: Raw counts, weighted proportions, and weighted P values were reported. Q1, 25^th^ quantile, Q3, 75^th^ quantile

^#^Differences between the two groups of journals were analyzed using Wilcoxon Rank Sum tests, Chi-Square tests or Fisher’s exact tests. Fisher’s exact tests were used when the count in certain cells was less than five

^*^Other regions included Africa, Asiatic region, Eastern Europe, Latin America, Middle East, and Pacific region

^¶^GAI guidelines of each journal were classified into four groups based on whether own and/or external guidelines were covered. Own guidelines refer to guidelines provided by journals and/or their publisher. External guidelines refer to guidelines provided by COPE, ICMJE, and WAME

**Fig. 2:**
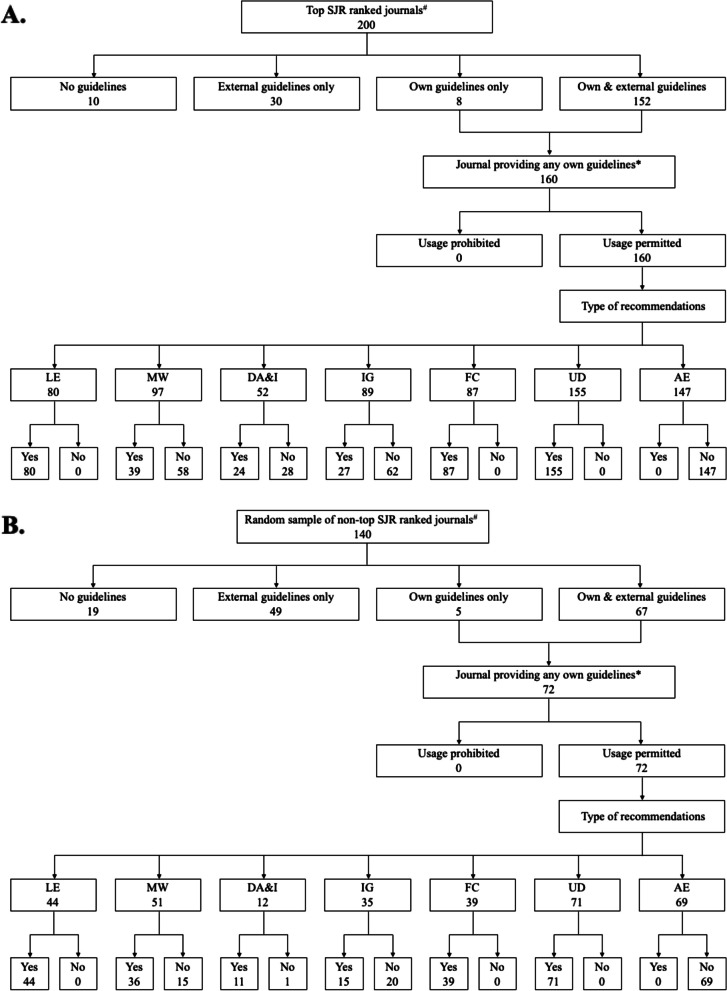
Coverage and type of recommendations for author guidelines among two groups of journals. The number in each cell represents the count of journals. Abbreviations: LE, language editing; MW, manuscript writing; DA&I, data analysis and interpretation; IG, image generating; FC, fact-checking; UD, usage documentation; AE, authorship eligibility. “Yes” or “No” was coded based on the item definitions provided in the Methods section. ^#^ The four groups of GAI usage guidelines coverage correspond to the counts presented in Table 2. ^*^ Journals providing any own guidelines correspond to the distribution of types of recommendations in Table 3

**Fig. 3:**
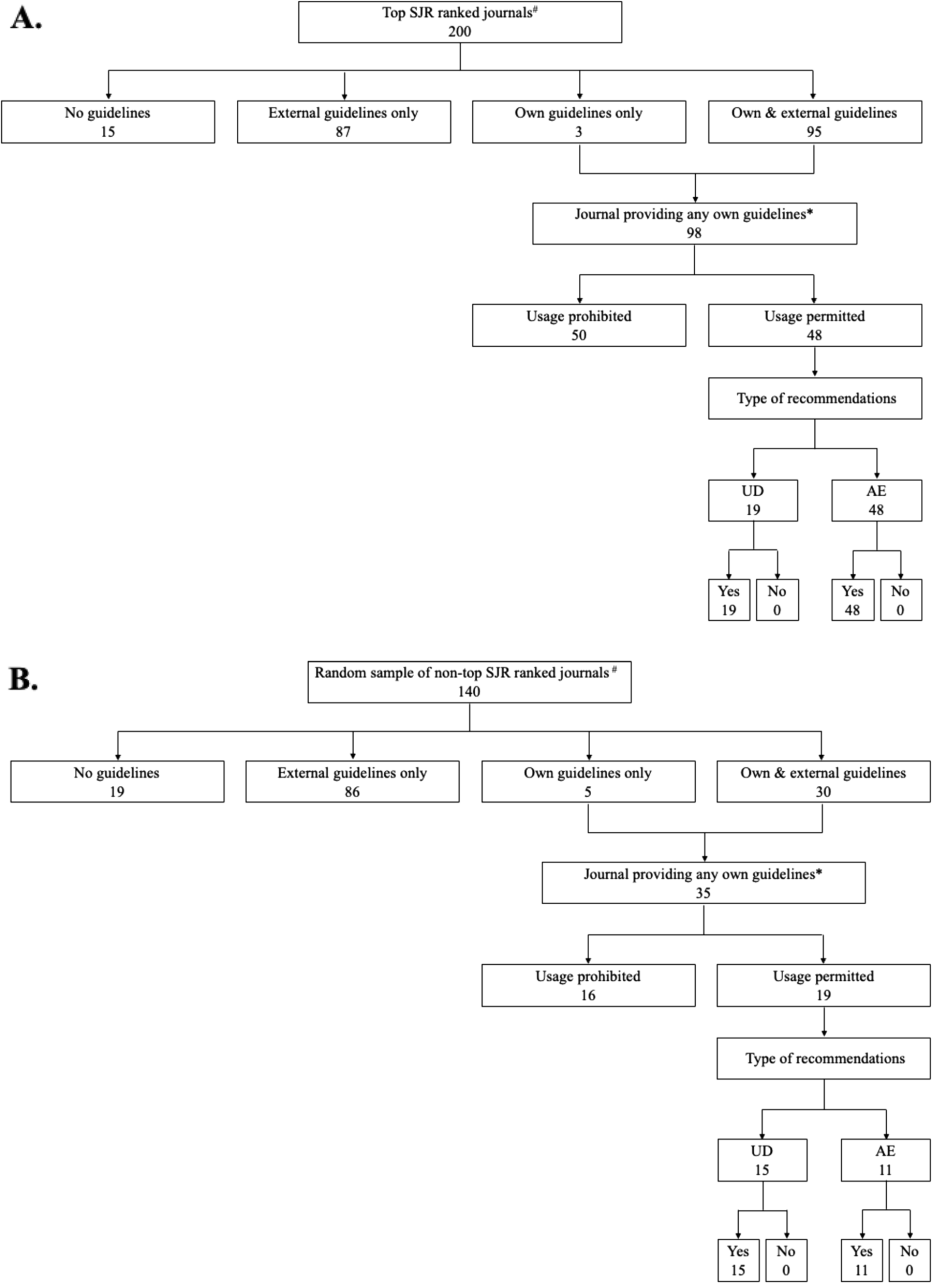
Coverage and type of recommendations for reviewer guidelines among two groups of journals. The number in each cell represents the count of journals. Abbreviations: LE, language editing; UD, usage documentation. “Yes” or “No” was coded based on the item definitions provided in the Methods section. ^#^ The four groups of GAI usage guidelines coverage correspond to the counts presented in Table 2. ^*^ Journals providing any own guidelines correspond to the distribution of types of recommendations in Table 3

A detailed summary of type of recommendations among journals providing any own GAI usage guidelines is presented for top SJR ranked journals and random sample of non-top SJR ranked journals in Table 3, including seven types of recommendations for authors and two types of recommendations for reviewers. For journals permitting GAI usage in author guidelines, both groups of journals are more likely to provide requirements on usage documentation (96.9% vs. 98.6%, *P* = 0.44) and author eligibility (91.9% vs. 95.8%, *P* = 0.27). However, both groups had lower percentages of journals providing recommendations for data analysis and interpretation, with the random sample of non-top SJR ranked journals having a significantly lower percentage (32.5% vs. 16.7%, *P* < 0.05). Around half of the journals in both groups provided recommendations for fact-checking (54.4% vs. 54.2%, *P* = 0.98). For journals permitting GAI usage for reviewers, top SJR ranked journals are more likely to require usage documentation (100.0% vs 57.9%, *P* < 0.01) while less likely to provide requirements on language editing (39.6% vs. 78.9%, *P* < 0.01) than the random sample of non-top SJR ranked journals. Among both groups of journals, the counts for each coding (i.e., “Yes” or “No”) of these recommendations were summarized for author guidelines (Fig. 2 A and B) and reviewer guidelines (Fig. 3 A and B). The two groups of journals shared a median of 5 on a scale of 0 to 7 for author guidelines and a median of 1 on a scale of 0 to 2 for reviewer guidelines regarding the number of recommendations.

**Table 3:** Distribution of type of recommendations among journals providing any own GAI usage guidelines

**A. Journals providing any own guidelines for authors**			
	**Top SJR ranked journals** **(*n*** **= 160)**^#^	**Random sample of non-top SJR ranked journals (*n* = 72)**^#^	***P*** value^*^
**Author guidelines, *n* (%)**			
Usage forbidden	0 (0.0)	0 (0.0)	-
Usage permitted	160 (100.0)	72 (100.0)	
**Type of recommendations among journals permitting GAI use,** ***n*** **(%)**^¶^			
Language editing	80 (50.0)	44 (61.1)	0.12
Manuscript writing	97 (60.6)	51 (70.8)	0.13
Data analysis and interpretation	52 (32.5)	12 (16.7)	< 0.05
Image generating	89 (55.6)	35 (48.6)	0.32
Fact-checking	87 (54.4)	39 (54.2)	0.98
Usage documentation	155 (96.9)	71 (98.6)	0.44
Authorship eligibility	147 (91.9)	69 (95.8)	0.27
**Number of recommendations, median (Q1, Q3)**			
Author guidelines (range: 0-7)	5 (3, 6)	5 (3, 6)	< 0.05

**B. Journals providing any own guidelines for reviewers**			
	**Top SJR ranked journals (*n*** **= 98)**^#^	**Random sample of non-top SJR ranked journals (*n*** **= 35)**^#^	***P*** value^*^
**Reviewer guidelines,** ***n*** **(%)**			
Usage forbidden	50 (51.0)	16 (45.7)	0.59
Usage permitted	48 (49.0)	19 (54.3)	
**Type of recommendations among journals permitting GAI use,** ***n*** **(%)**^¶^			
Language editing	19 (39.6)	15 (78.9)	< 0.01
Usage documentation	48 (100.0)	11 (57.9)	< 0.01
**Number of recommendations, median (Q1, Q3)**			
Reviewer guidelines (range: 0-2)	1 (1, 2)	1 (1, 2)	< 0.01

Footnote: Raw counts, weighted proportions, and weighted P values were reported. Q1, 25^th^ quantile, Q3, 75^th^ quantile

^#^Journals providing any own guidelines were included (Own guidelines only and Own & external guidelines in Table 2)

^*^Differences between the two groups of journals were analyzed using Wilcoxon Rank Sum tests, t-tests, or Chi-Square tests

^¶^The percentage was calculated using the number of “usage permitted” journals as the denominator

Relationships between journal characteristics and the coverage of GAI usage guidelines among top SJR ranked journals (Table 4) differed from those among the random sample of non-top SJR ranked journals (Table 5). Among top SJR ranked journals, no significant associations were observed between journal characteristics and the coverage of either author or reviewer guidelines. However, among the random sample of non-top SJR ranked journals, a higher SJR score was positively associated with providing GAI usage guidelines for both authors (all *P* < 0.01) and reviewers (all *P* < 0.01). Table 6 showed the relationships between journal characteristics and the coverage of GAI usage guidelines after pooling two groups of journals. Specifically, higher SJR scores were associated with the increased coverage of author guidelines (all *P* < 0.05) and with the coverage of own and external guidelines for reviewer guidelines (*P* < 0.01).

**Table 4:** Multinomial logistic regression examining the relationship between journal characteristics and the coverage of GAI usage guidelines among the 200 top SJR ranked journals

	Top SJR ranked journals (***n*** = 200)									
	**No guidelines**	**External guidelines only**			**Own guidelines only**			**Own and external guidelines**		
**Journal characteristics**		**Coef.**	**95% CI**	***P*** **value**	**Coef.**	**95% CI**	***P*** **value**	**Coef.**	**95% CI**	***P*** **value**
		**Coverage of author guidelines**								
SJR score	Ref.	0.47	(-0.05, 0.98)	0.08	0.38	(-0.18, 0.94)	0.18	0.45	(-0.07, 0.96)	0.09
Region										
Northern America	Ref.	-	-	-	-	-	-	-	-	-
Western Europe	Ref.	-0.92	(-2.49,0.64)	0.25	0.22	(-1.69, 2.13)	0.82	0.51	(-0.84, 1.87)	0.46
Other regions^#^	Ref.	-1.48	(-4.41, 1.46)	0.32	-12.17	(-976.04, 951.70)	0.98	-12.12	(-244.87, 220.64)	0.92
Focus area										
General medicine	Ref.	-	-	-	-	-	-	-	-	-
Specialty areas	Ref.	0.78	(-0.74, 2.31)	0.31	1.54	(-0.91, 3.99)	0.22	1.08	(-0.25, 2.41)	0.11
		**Coverage of reviewer guidelines**								
SJR score	Ref.	0.18	(-0.08, 0.45)	0.18	0.10	(-0.40, 0.60)	0.69	0.24	(-0.03, 0.51)	0.08
Region										
Northern America	Ref.	-	-	-	-	-	-	-	-	-
Western Europe	Ref.	-0.47	(-1.61, 0.66)	0.41	-0.69	(-3.31, 1.93)	0.60	0.58	(-0.54, 1.71)	0.31
Other regions^#^	Ref.	-2.02	(-4.91, 0.86)	0.17	-6.24	(-89.26, 76.79)	0.88	-9.89	(-134.78, 115.01)	0.88
Focus area										
General medicine	Ref.	-	-	-	-	-	-	-	-	-
Specialty areas	Ref.	0.45	(-0.73, 1.63)	0.45	7.26	(-53.09, 67.61)	0.81	1.06	(-0.15, 2.28)	0.09

Footnote: Weighted regression results were reported

^#^Other regions included Africa, Asiatic region, Eastern Europe, Latin America, Middle East, and Pacific region

**Table 5:** Multinomial logistic regression examining the relationship between journal characteristics and the coverage of GAI usage guidelines among the random sample of 140 non-top SJR ranked journals

	**Random sample of non-top SJR ranked journals (*n*** **= 140)**									
	**No guidelines**	**External guidelines only**			**Own guidelines only**			**Own & external guidelines**		
**Journal characteristics**		**Coef.**	**95% CI**	***P*** **value**	**Coef.**	**95% CI**	***P*** **value**	**Coef.**	**95% CI**	***P*** **value**
		**Coverage of author guidelines**								
SJR score	Ref.	7.40	(2.41, 12.39)	< 0.01	10.36	(5.13, 15.59)	< 0.01	10.02	(4.96, 15.10)	< 0.01
Region										
Northern America	Ref.	-	-	-	-	-	-	-	-	-
Western Europe	Ref.	0.11	(-1.46, 1.68)	0.89	1.21	(-1.38, 3.80)	0.36	0.52	(-0.87, 1.90)	0.46
Other regions^#^	Ref.	0.65	(-0.67, 1.97)	0.33	-0.59	(-3.56, 2.39)	0.70	-1.19	(-2.49, 0.10)	0.07
Focus area										
General medicine	Ref.	-	-	-	-	-	-	-	-	-
Specialty areas	Ref.	0.06	(-1.16, 1.29)	0.92	0.32	(-2.10, 2.74)	0.79	0.36	(-0.84, 1.56)	0.56
		**Coverage of reviewer guidelines**								
SJR score	Ref.	8.27	(3.37, 13.18)	< 0.01	9.48	(4.40, 14.57)	< 0.01	9.27	(4.32, 14.21)	< 0.01
Region										
Northern America	Ref.	-	-	-	-	-	-	-	-	-
Western Europe	Ref.	0.15	(-1.24, 1.54)	0.83	1.21	(-1.38, 3.80)	0.36	1.00	(-0.55, 2.54)	0.21
Other regions^#^	Ref.	-0.16	(-1.36, 1.05)	0.80	-0.59	(-3.56, 2.39)	0.70	-0.74	(-2.28, 0.80)	0.35
Focus area										
General medicine	Ref.	-	-	-	-	-	-	-	-	-
Specialty areas	Ref.	0.20	(-0.96, 1.36)	0.74	0.32	(-2.10, 2.75)	0.79	0.32	(-1.05, 1.69)	0.65

Footnote: Weighted regression results were reported

^#^Other regions included Africa, Asiatic region, Eastern Europe, Latin America, Middle East, and Pacific region

**Table 6:** Multinomial logistic regression examining the relationship between journal characteristics and the coverage of GAI usage guidelines after pooling two groups of journals (*n* = 340)

	**No guidelines**	**External guidelines only**			**Own guidelines only**			**Own & external guidelines**		
**Journal characteristics**		**Coef.**	**95% CI**	***P*** **value**	**Coef.**	**95% CI**	***P*** **value**	**Coef.**	**95% CI**	***P*** **value**
		**Coverage of author guidelines **								
SJR score	Ref.	0.28	(0.07, 0.49)	< 0.05	0.32	(0.07, 0.57)	< 0.05	0.37	(0.16, 0.57)	< 0.01
Region										
Northern America	Ref.	-	-	-	-	-	-	-	-	-
Western Europe	Ref.	-0.50	(-1.59, 0.60)	0.38	0.50	(-0.96, 1.97)	0.50	0.49	(-0.47, 1.44)	0.32
Other regions^#^	Ref.	0.51	(-0.97, 1.04)	0.95	-1.61	(-3.92, 0.71)	0.17	-1.98	(-3.04, -0.92)	< 0.01
Focus area										
General medicine	Ref.	-	-	-	-	-	-	-	-	-
Specialty areas	Ref.	0.33	(-0.61, 1.28)	0.49	0.89	(-0.81, 2.59)	0.31	0.65	(-0.21, 1.52)	0.14
		**Coverage of reviewer guidelines **								
SJR score	Ref.	0.14	(-0.01, 0.30)	0.06	0.06	(-0.24, 0.36)	0.69	0.26	(0.11, 0.42)	< 0.01
Region										
Northern America	Ref.	-	-	-	-	-	-	-	-	-
Western Europe	Ref.	-0.24	(-1.12, 0.64)	0.59	0.33	(-1.37, 2.03)	0.70	0.69	(-0.20, 1.58)	0.13
Other regions^#^	Ref.	-0.52	(-1.45, 0.40)	0.27	-0.92	(-3.33, 1.49)	0.46	-1.74	(-2.94, -0.54)	< 0.01
Focus area										
General medicine	Ref.	-	-	-	-	-	-	-	-	-
Specialty areas	Ref.	0.31	(-0.51, 1.13)	0.46	1.05	(-1.17, 3.27)	0.35	0.77	(-0.12, 1.65)	0.09

Footnote: Weighted regression results were reported

^#^Other regions included Africa, Asiatic region, Eastern Europe, Latin America, Middle East, and Pacific region

The type of recommendations of guidelines was detailed among top SJR ranked journals and the random sample of non-top SJR ranked journals that permit GAI usage (Additional file 1: Supplementary Figs. 1 and 2). For author guidelines, a consensus emerged on four recommendations whenever provided: permitting language editing, requiring fact-checking, mandating usage documentation, and prohibiting AI authorship. However, disagreements were observed about whether GAI can be used for writing manuscripts, analyzing and interpreting data, and generating images. For reviewer guidelines, there were disagreements on permitting GAI usage and permitting language editing. Examples were provided for each type of recommendation for author and reviewer guidelines (Additional file 1: Supplementary Table 5). Among external guidelines, the ICMJE guideline was the most frequently referred to (Additional file 1: Supplementary Table 6). Among the random sample of non-top SJR ranked journals, similar patterns of recommendations were observed except that a lower proportion of journals provided specific types of recommendations.

Relationships between journal characteristics and number of recommendations of author and reviewer guidelines were examined among the two groups of journals (Additional file 1: Supplementary Table 7). Across both groups of journals, none of the three journal characteristics were associated with the number of recommendations of usage guidelines. Consistent results were observed for the relationships between journal characteristics and number of recommendations of author and reviewer guidelines when pooling the two groups of journals (Additional file 1: Supplementary Table 8). Additionally, the sensitivity analysis by adding one journal without an SJR score yielded almost identical findings (Additional file 1: Supplementary Tables 9 and 10). The number of recommendations for authors was 5 for all three external guidelines (COPE, ICMJE, and WAME), with the specific items being largely similar across these guidelines (Additional file 1: Supplementary Table 11). Notably, none of these guidelines included recommendations on language editing for either authors or reviewers.

## Discussion

Our study systematically examined GAI usage guidelines for scholarly publishing across medical journals, including author guidelines, reviewer guidelines, and references to external guidelines. The majority of top SJR ranked journals and the random sample of non-top SJR ranked journals provided GAI usage guidelines for authors and reviewers. This highlights medical journals’ proactive role in implementing GAI guidelines, likely due to their strong commitment to maintaining high standards in scholarly publishing [44]. The median of number of recommendations for author guidelines and reviewer guidelines were identical between the two groups of journals, indicating that once guidelines are adopted, the level of comprehensiveness tends to be largely similar. Despite this, these guidelines often lacked comprehensive coverage of all types of academic practices. The high proportion of both journal groups referred to external guidelines showed the critical role of these external standards in fostering ethical practices of GAI usage. Among the random sample of non-top SJR ranked journals, we also identified SJR scores that were associated with the coverage of different guidelines. Together, our results suggest that collaborative efforts are needed for explorations and improvements of GAI usage guidelines in scholarly publishing.

This study presents a timely, systematic, and in-depth evaluation of GAI usage guidelines across both the top and non-top SJR ranked medical journals. The relationships between journals characteristics and the coverage of such guidelines have been examined. Among non-top SJR ranked journals, a journal with a higher SJR score was more likely to provide GAI usage guidelines for both authors and reviewers. This finding is consistent with the previous study that showed a positive association between citation metrics and the inclusion of ethical topics in journal guidelines [44]. One possible reason may be that lower-ranked journals lack the resources or awareness to provide guidelines, which could lead to inconsistencies in GAI usage guidelines across different tiers of journals. This finding underscores the need for attention to journals with lower rankings.

In comparison to a previous study [40], which reported that 86% of top journals provided author guidelines, our study found a similar proportion (80%) among top SJR ranked journals. Despite the high proportion of guideline coverage, top SJR ranked journals were more likely to provide author guidelines than non-top SJR ranked journals. This finding aligns with our expectations because of top SJR ranked journals’ initiative role in establishing standards on the use of emerging technologies such as GAI. These better coverages can be attributed to several factors. They include more accessible advanced AI technology, stringent regulatory environments, and robust scholarly communication networks [50–52].

We did not observe significant regional differences in GAI guideline coverage, possibly because the overall coverage of guidelines was relatively high across all regions. This observation contrasts with previous studies that have identified regional differences as a key factor in how research integrity topics are addressed in author guidelines [35]. One potential explanation is that GAI guidelines are being adopted more uniformly across regions due to the global nature of AI’s impact on scholarly publishing.

Both the top SJR ranked journals and non-top SJR ranked journals had a median of five recommendations for their author guidelines out of a seven. While some types of recommendations were provided for authors, there is still considerable room for enhancement. For example, both groups had lower percentages of journals providing recommendations for data analysis and interpretation and fact-checking. Data analysis and interpretation are fundamental to the reliability of research findings and fact-checking is essential to ensure the accuracy of the produced content. Lack of guidance in these critical areas may raise significant concerns such as inappropriate research conduct and the dissemination of false or biased information. A greater comprehensiveness can help authors adhere to ethical standards and prevent potential misconduct, including plagiarism or misrepresentation of AI-generated content as human work. Our findings showed that both top and non-top SJR ranked journals have room for improvement.

Across journals, consensus has been achieved for four of the seven recommendations examined in author guidelines, and disagreements were observed for three recommendations related to content generating applications. The remarkable capacities of GAI justify its usage in scholarly writing, especially for language editing [4, 53–55]. However, its risks threatening research integrity require authors to conduct fact-checking and usage documentation but not to grant such tools authorship [56]. These agreements show how the scientific community is embracing GAI in a cautious way. For example, the emphasis on fact-checking and usage documentation is necessary for preventing fabrication and potential biases and promoting transparency and trust [57].

Notably, journals differ in their instructions on where and how to disclose GAI usage. Some journals may not specify a particular location for the declaration or mention it generally as part of the submitted work. Others may provide more detailed guidance, specifying that GAI usage should be disclosed in the methods section, cover letter, or acknowledgment section. Additionally, while some journals require authors to report any use of GAI, other journals permit authors not to do so if such tools were solely used for language editing. This may be explained by journals’ different interpretations of GAI applications. Together with two other recent studies showing inconsistencies in what to disclose of GAI usage, a need is highlighted surrounding standardization of GAI usage disclosure and documentation [40, 41, 58]. More importantly, journals held different and even opposite stances for content-generating applications, including manuscript writing, data analysis and interpretation, and image generating. This is due to concern centered on plagiarism because it is challenging to trace and verify the originality of AI-generated content [59, 60].

As the first study that systematically examined GAI usage guidelines for reviewers, we observed disagreements on recommendations regarding usage permission across journals. We interpreted this disagreement as journals’ different stances on how to protect the confidentiality of manuscripts [8, 61]. Some journals specified conditions in which GAI can be used. For example, they required reviewers to obtain permission from authors and editors, to confirm that manuscripts shall not be uploaded as training data, and provide authors with the choice to opt for or against a GAI-assisted review process [30, 32].

Around 90% of top SJR ranked journals and 80% of the random sample of non-top SJR ranked journals have referred to external guidelines on GAI usage formulated by ICMJE, COPE, or WAWE. This underscores a collective endeavor to establish ethical benchmarks for GAI usage in scholarly publishing. Across the three external guidelines, all permitted authors and reviewers to use GAI. For the seven types of recommendations for authors, all guidelines addressed three items: prohibiting AI authorship, mandating usage documentation, and requiring fact-checking. While there are minimal variations among the guidelines, they are largely similar in their recommendations. Notably, language editing for authors and reviewers was not covered in any of the three external guidelines. It is possible that this omission arises from the perception that language editing, which is often managed by specialized services, is a well-established and safe practice, rendering explicit recommendations unnecessary [4, 55]. Another possibility is the perception that language editing poses fewer concerns related to misinformation and bias compared to content generation [56]. However, some organizations have adopted a stricter standard to promote transparency in GAI usage. For instance, the European Association of Science Editors (EASE) recommends that authors declare their use of AI for language editing in the Acknowledgements section or in a dedicated AI declaration section; In cases where this does not need to be declared, explicit recommendation should be provided to specify [62].

We observed overlaps and discrepancies between journal’s own guidelines and external guidelines. For author guidelines, we identified overlaps in usage permissions, with both journal’s own and external guidelines permitting GAI usage. Among the seven types of recommendations, our analysis revealed three areas of overlap: fact-checking, usage documentation, and authorship eligibility, where both journal’s own and external guidelines aligned whenever these aspects were mentioned.

However, language editing presented as one of the discrepancies. While many journals permitted its use for authors, external guidelines did not explicitly address this practice. Other discrepancies also included manuscript writing, data analysis and interpretation, and image generating. For example, different journals may have formulated opposing recommendations for these items in their guidelines. For reviewer guidelines, discrepancies between journal’s own and external guidelines were pronounced in terms of usage permission. While journal’s own guidelines showed opposing recommendations, all three external guidelines permitted GAI usage for reviewers. Additionally, some journals provided recommendations on language editing for peer review, which was not covered by external guidelines.

We identified three main aspects of GAI usage guidelines that need urgent improvements. First, while most medical journals provided their own GAI usage guidelines or referenced external guidelines, these guidelines often lacked comprehensive coverage of all types and deserved improvement. Details of the guidelines should be specified to avoid confusion, especially for content-generating applications. Our systematic evaluation of available GAI usage guidelines provides a roadmap to clarify expectations on proper GAI practices. Second, disagreements identified across guidelines warrant special attention. Although these disagreements highlight the continuously evolving use of GAI, it is possible to establish a framework of proper practices and specify recommendations under different scenarios. For example, journals may consider standardizing usage documentation by integrating questions into the manuscript submission system; ascertaining whether GAI was used, and if so, detailing specific purposes and ensuring accountability. Third, given the rapid evolution of GAI systems, scientific communities have recommended the adoption of “living” GAI usage guidelines. These guidelines will undergo continuous revision and adaptation either monthly or every three to six months [63]. Regular updates to author and reviewer guidelines are necessary to keep pace with evolving ethical standards, technological advancements, and best practices in scholarly publishing [33]. This dynamic approach ensures that guidelines remain relevant and effective in addressing the challenges posed by new AI developments.

Our study has several strengths. It pioneered a systematic and quantitative examination of GAI usage guidelines across top SJR ranked journals and a random sample of non-top SJR ranked journals, which provides an overview of the current regulations in scholarly publishing. It also synthesized existing GAI usage guidelines for both authors and reviewers and established a systematic checklist. We further provided recommendations aimed at improving existing GAI usage guidelines for scholarly publishing and promoting the proper use of GAI tools. While our study focused on medical journals, we encourage broadening the scope of this type of research to include other disciplines. This will provide a more comprehensive understanding of the scholarly publishing landscape across various fields and help identify discipline-specific challenges and best practices. Such expanded research efforts would contribute to the development of more nuanced and effective guidelines that address the diverse needs of the academic community.

Our study also has limitations. First, we only focused on publicly available information for reviewer guidelines due to the availability of related data. This is because reviewer guidelines in some journals may be accessible only through submission systems or via invitation emails. Incorporating such information in future research may give a more complete overview of GAI usage for reviewers. Similarly, for usage documentation, our analysis was based solely on publicly available information from the journal or publisher’s official website. Future research could gain deeper insights by conducting an in-depth review of submission systems to capture a more comprehensive understanding of GAI usage documentation. Second, the checklist of recommendations we identified and used to calculate the number of recommendations may not reflect equal importance. As GAI tools continue to develop rapidly, the relative significance of individual recommendations may also shift over time. While our checklist was designed to offer a foundational overview of GAI usage guidelines, it is important to interpret the number of recommendations with caution. Additionally, as a cross-sectional study, our data was retrieved from a predefined list of journals on a specific 7-day period. Future studies can access the historical records of the guidelines to provide insights about how these guidelines are developed and adapted over time. Third, while we used SJR score as the primary metric for journal selection, we acknowledge the importance of incorporating other metrics, such as impact factor, to provide a more comprehensive analysis. Future studies may explore different journal ranking metrics to further validate and expand on our findings.

## Conclusions

Although most medical journals provided their own GAI usage guidelines or referenced external guidelines, some recommendations remained unspecified. For example, many journals did not address whether AI can be used for data analysis and interpretation, or whether authors should conduct fact-checking. Providing recommendations on these aspects can be helpful in guiding authors on the appropriate use of GAI tools in their research. Additionally, journals with lower SJR scores were less likely to provide guidelines, indicating a potential gap that warrants attention. Collaborative efforts are needed to develop comprehensive recommendations that better guide authors and reviewers in GAI usage.

## Supplementary Information

Additional file 1: Supplementary Table 1. STROBE Statement—Checklist of items that should be included in cross-sectional studies. Supplementary Table 2. Journal characteristics and information sources of generative artificial intelligence (GAI) usage guidelines of the 200 top SJR ranked journals. Supplementary Table 3. Journal characteristics and information sources of GAI usage guidelines of the random sample of 140 non-top SJR ranked journals. Supplementary Table 4. External GAI usage guidelines and their recommendations. Supplementary Table 5. Examples of type of recommendations for author and reviewer guidelines. Supplementary Table 6. Coverage of external GAI usage guidelines among top SJR ranked journals and random sample of non-top SJR ranked journals. Supplementary Table 7. Linear regression analysis of the relationship between journal characteristics and number of recommendations for GAI usage guidelines among the 200 top SJR ranked journals and the random sample of 140 non-top SJR ranked journals separately. Supplementary Table 8. Linear regression analysis of the relationship between journal characteristics and the number of recommendations of GAI usage guidelines after pooling two groups of journals (*n* = 340). Supplementary Table 9. Multinomial logistic regression examining the relationship between journal characteristics and and the coverage of GAI usage guidelines among the random sample of 141 non-top SJR ranked journals (after adding one random journal without SJR score to Table 5). Supplementary Table 10. Multinomial logistic regression examining the relationship between journal characteristics and the coverage of GAI usage guidelines after pooling two groups of journals (*n* = 341) (after adding one random journal without SJR score to Table 6). Supplementary Table 11. Number of recommendations across external GAI usage guidelines. Supplementary Fig. 1. Type of recommendations across different GAI usage guidelines among top SJR ranked journals. Supplementary Fig. 2. Type of recommendations across different GAI usage guidelines among random sample of non-top SJR ranked journals.

## Data Availability

All our data and code used for data analysis, along with relevant statistical outputs, has been deposited in the GitHub repository: https://github.com/simuh34/GAI-usage-guidelines-across-medical-journals.

## Abbreviations

GAI: Generative artificial intelligence
SJR: SCImago Journal Rank
ICMJE: The International Committee of Medical Journal Editors
COPE: The Committee on Publication Ethics
WAME: The World Association of Medical Editors
LE: Language editing
MW: Manuscript writing
DA&I: Data analysis and interpretation
IG: Image generating
FC: Fact-checking
UD: Usage documentation
AE: Authorship eligibility
