# Supplementary material for: Generative artificial intelligence (GAI) usage guidelines for scholarly publishing: a cross-sectional study of medical journals
Source: BMC Med. 2025 Feb 11;23:77. doi: 10.1186/s12916-025-03899-1 (PMC11816781; doi:10.1186/s12916-025-03899-1)
Supplement: Supplementary file 1 — Additional file 1: Supplementary Table 1. STROBE Statement—Checklist of items that should be included in cross-sectional studies. Supplementary Table 2. Journal characteristics and information sources of generative artificial intelligence (GAI) usage guidelines of the 200 top SJR ranked journals. Supplementary Table 3. Journal characteristics and information sources of GAI usage guidelines of the random sample of 140 non-top SJR ranked journals. Supplementary Table 4. External GAI usage guidelines and their recommendations. Supplementary Table 5. Examples of type of recommendations for author and reviewer guidelines. Supplementary Table 6. Coverage of external GAI usage guidelines among top SJR ranked journals and random sample of non-top SJR ranked journals. Supplementary Table 7. Linear regression analysis of the relationship between journal characteristics and number of recommendations for GAI usage guidelines among the 200 top SJR ranked journals and the random sample of 140 non-top SJR ranked journals separately. Supplementary Table 8. Linear regression analysis of the relationship between journal characteristics and the number of recommendations of GAI usage guidelines after pooling two groups of journals (n = 340). Supplementary Table 9. Multinomial logistic regression examining the relationship between journal characteristics and and the coverage of GAI usage guidelines among the random sample of 141 non-top SJR ranked journals (after adding one random journal without SJR score to Table 5). Supplementary Table 10. Multinomial logistic regression examining the relationship between journal characteristics and the coverage of GAI usage guidelines after pooling two groups of journals (n = 341) (after adding one random journal without SJR score to Table 6). Supplementary Table 11. Number of recommendations across external GAI usage guidelines. Supplementary Fig. 1. Type of recommendations across different GAI usage guidelines among top SJR ranked journals. Supple [file 12916_2025_3899_MOESM1_ESM.docx]

**Supplementary Material**

**Table of Contents**

**Supplementary Table 1. STROBE Statement—Checklist of items that should be included in *cross-sectional studies***

**Supplementary Table 2. Journal characteristics and information sources of generative artificial intelligence (GAI) usage guidelines of the 200 top SJR ranked journals**

**Supplementary Table 3. Journal characteristics and information sources of GAI usage guidelines of the random sample of 140 non-top SJR ranked journals**

**Supplementary Table 4. External GAI usage guidelines and their recommendations**

**Supplementary Table 5. Examples of type of recommendations for author and reviewer guidelines**

**Supplementary Table 6. Coverage of external GAI usage guidelines among top SJR ranked journals and random sample of non-top SJR ranked journals**

**Supplementary Table 7. Linear regression analysis of the relationship between journal characteristics and number of recommendations for GAI usage guidelines among the 200 top SJR ranked journals and the random sample of 140 non-top SJR ranked journals separately**

**Supplementary Table 8. Linear regression analysis of the relationship between journal characteristics and the number of recommendations of GAI usage guidelines after pooling two groups of journals (n = 340)**

**Supplementary Table 9. Multinomial logistic regression examining the relationship between journal characteristics and the coverage of GAI usage guidelines among the random sample of 141 non-top SJR ranked journals (after adding one random journal without SJR score to Table 5)**

**Supplementary Table 10. Multinomial logistic regression examining the relationship between journal characteristics and the coverage of GAI usage guidelines after pooling two groups of journals (n = 341) (after adding one random journal without SJR score to Table 6)**

**Supplementary Table 11. Number of recommendations across external GAI usage guidelines**

**Supplementary Figure 1. Type of recommendations across different GAI usage guidelines among top SJR ranked journals**

**Supplementary Figure 2. Type of recommendations across different GAI usage guidelines among random sample of non-top SJR ranked journals**

**Supplementary Table 1. STROBE Statement—Checklist of items that should be included in *cross-sectional studies***

|  | Item No | Recommendation | Page |
| --- | --- | --- | --- |
| **Title and abstract** | 1 | (*a*) Indicate the study’s design with a commonly used term in the title or the abstract | 1 |
|  |  | (*b*) Provide in the abstract an informative and balanced summary of what was done and what was found | 1 |
| Introduction | | |  |
| Background/rationale | 2 | Explain the scientific background and rationale for the investigation being reported | 1 |
| Objectives | 3 | State specific objectives, including any prespecified hypotheses | 2 |
| Methods | | |  |
| Study design | 4 | Present key elements of study design early in the paper | 3 |
| Setting | 5 | Describe the setting, locations, and relevant dates, including periods of recruitment, exposure, follow-up, and data collection | 3 |
| Participants | 6 | (*a*) Give the eligibility criteria, and the sources and methods of selection of participants | 3 |
| Variables | 7 | Clearly define all outcomes, exposures, predictors, potential confounders, and effect modifiers. Give diagnostic criteria, if applicable | 4 |
| Data sources/ measurement | 8* | For each variable of interest, give sources of data and details of methods of assessment (measurement). Describe comparability of assessment methods if there is more than one group | 6 |
| Bias | 9 | Describe any efforts to address potential sources of bias | NA |
| Study size | 10 | Explain how the study size was arrived at | 3 |
| Quantitative variables | 11 | Explain how quantitative variables were handled in the analyses. If applicable, describe which groupings were chosen and why | 3 |
| Statistical methods | 12 | (*a*) Describe all statistical methods, including those used to control for confounding | 6 |
|  |  | (*b*) Describe any methods used to examine subgroups and interactions | 3, 4 |
|  |  | (*c*) Explain how missing data were addressed | 4 |
|  |  | (*d*) If applicable, describe analytical methods taking account of sampling strategy | 6 |
|  |  | (*e*) Describe any sensitivity analyses | NA |
| Results | | |  |
| Participants | 13* | (a) Report numbers of individuals at each stage of study—eg numbers potentially eligible, examined for eligibility, confirmed eligible, included in the study, completing follow-up, and analysed | 3 |
|  |  | (b) Give reasons for non-participation at each stage | NA |
|  |  | (c) Consider use of a flow diagram | 3 |
| Descriptive data | 14* | (a) Give characteristics of study participants (eg demographic, clinical, social) and information on exposures and potential confounders | 3 |
|  |  | (b) Indicate number of participants with missing data for each variable of interest | 6 |
| Outcome data | 15* | Report numbers of outcome events or summary measures | 6-8 |
| Main results | 16 | (*a*) Give unadjusted estimates and, if applicable, confounder-adjusted estimates and their precision (eg, 95% confidence interval). Make clear which confounders were adjusted for and why they were included | 7 |
|  |  | (*b*) Report category boundaries when continuous variables were categorized | 7 |
|  |  | (*c*) If relevant, consider translating estimates of relative risk into absolute risk for a meaningful time period | 13 |
| Other analyses | 17 | Report other analyses done—eg analyses of subgroups and interactions, and sensitivity analyses | 7, 8 |
| Discussion | | |  |
| Key results | 18 | Summarise key results with reference to study objectives | 8, 9 |
| Limitations | 19 | Discuss limitations of the study, taking into account sources of potential bias or imprecision. Discuss both direction and magnitude of any potential bias | 12, 13 |
| Interpretation | 20 | Give a cautious overall interpretation of results considering objectives, limitations, multiplicity of analyses, results from similar studies, and other relevant evidence | 9-11 |
| Generalisability | 21 | Discuss the generalisability (external validity) of the study results | 12 |
| Other information | | |  |
| Funding | 22 | Give the source of funding and the role of the funders for the present study and, if applicable, for the original study on which the present article is based | 1 |

*NA: not applicable

**Supplementary Table 2. Journal characteristics and information sources of generative artificial intelligence (GAI) usage guidelines of the 200 top SJR ranked journals**

| **ID** | **Journal name** | **Publisher** | **SJR score** | **Region** | **Focus area** | **Websites** |
| --- | --- | --- | --- | --- | --- | --- |
| 1 | Ca-A Cancer Journal for Clinicians | Wiley | 86.09 | Northern America | Speciality areas | https://acsjournals.onlinelibrary.wiley.com/journal/15424863; https://acsjournals.onlinelibrary.wiley.com/hub/journal/15424863/homepage/forauthors.html |
| 2 | New England Journal of Medicine | Massachussetts Medical Society | 26.02 | Northern America | General medicine | https://www.nejm.org/about-nejm/editorial-policies; https://www.nejm.org/media-center/publication-process |
| 3 | Nature Medicine | Nature Publishing Group | 24.69 | Western Europe | General medicine | https://www.nature.com/nm/editorial-policies; https://www.nature.com/nm/editorial-policies/peer-review |
| 4 | MMWR Recommendations and Reports | Centers for Disease Control and Prevention (CDC) | 23.96 | Northern America | Speciality areas | https://www.cdc.gov/mmwr/author_guide.html |
| 5 | Nature Reviews Cancer | Nature Publishing Group | 20.98 | Western Europe | Speciality areas | https://www.nature.com/nrc/for-authors/preparing-your-submission; https://www.nature.com/nature-portfolio/editorial-policies/ai |
| 6 | Nature Reviews Genetics | Nature Publishing Group | 19.45 | Western Europe | Speciality areas | https://www.nature.com/nrg/for-authors/preparing-your-submission https://www.nature.com/nature-portfolio/editorial-policies/ai; https://www.nature.com/nature-portfolio/editorial-policies/ai |
| 7 | Nature Reviews Drug Discovery | Nature Publishing Group | 19.42 | Western Europe | General medicine | https://www.nature.com/nrd/for-authors/preparing-your-submission https://www.nature.com/nature-portfolio/editorial-policies/ai; https://www.nature.com/nature-portfolio/editorial-policies/ai |
| 8 | Nature Reviews Immunology | Nature Publishing Group | 18.14 | Western Europe | Speciality areas | https://www.nature.com/nri/for-authors/preparing-your-submission https://www.nature.com/nature-portfolio/editorial-policies/ai; https://www.nature.com/nature-portfolio/editorial-policies/ai |
| 9 | Morbidity and Mortality Weekly Report | Department of Health and Human Services | 16.79 | Northern America | Speciality areas | https://www.cdc.gov/mmwr/author_guide.html |
| 10 | Annual Review of Immunology | Annual Reviews | 15.26 | Northern America | Speciality areas | https://www.annualreviews.org/pb-assets/authors%20assets/authorhandbook-numbered.pdf |
| 11 | MMWR Surveillance Summaries | Centers for Disease Control and Prevention (CDC) | 15.18 | Northern America | Speciality areas | https://www.cdc.gov/mmwr/author_guide.html |
| 12 | Nature Reviews Clinical Oncology | Nature Publishing Group | 14.81 | Western Europe | Speciality areas | https://www.nature.com/nrclinonc/for-authors/preparing-your-submission; https://www.nature.com/nature-portfolio/editorial-policies/ai |
| 13 | Immunity | Cell Press | 14.80 | Northern America | Speciality areas | https://www.cell.com/immunity/authors; https://www.cell.com/reviewers |
| 14 | The Lancet | Elsevier | 14.61 | Western Europe | General medicine | https://www.thelancet.com/pb/assets/raw/Lancet/authors/tl-info-for-authors-1717152770277.pdf; https://www.thelancet.com/peer-review |
| 15 | World Psychiatry | Wiley | 14.31 | Northern America | Speciality areas | https://onlinelibrary.wiley.com/page/journal/20515545/homepage/forauthors.html; https://authorservices.wiley.com/ethics-guidelines/index.html |
| 16 | Physiological Reviews | American Physiological Society | 13.69 | Northern America | Speciality areas | https://journals.physiology.org/editorial-policies#AI-policy |
| 17 | MMWR supplements | Epidemiology Program Office | 13.47 | Northern America | General medicine | https://www.cdc.gov/mmwr/author_guide.html |
| 18 | Cancer Cell | Cell Press | 12.58 | Northern America | Speciality areas | https://www.cell.com/cancer-cell/authors |
| 19 | The Lancet Oncology | Elsevier | 12.27 | Western Europe | Speciality areas | https://www.thelancet.com/publishing-excellence; https://www.thelancet.com/peer-review |
| 20 | Annals of Oncology | Elsevier | 11.95 | Western Europe | Speciality areas | https://www.sciencedirect.com/journal/annals-of-oncology/publish/guide-for-authors |
| 21 | Nature Reviews Disease Primers | Nature Publishing Group | 11.39 | Western Europe | General medicine | https://www.nature.com/nrdp/for-authors/preparing-your-submission; https://www.nature.com/nature-portfolio/editorial-policies/ai; https://www.nature.com/nature-portfolio/editorial-policies/ai |
| 22 | The Lancet Respiratory Medicine | Elsevier | 11.20 | Western Europe | Speciality areas | https://www.thelancet.com/pb/assets/raw/Lancet/authors/tlrm-info-for-authors-1717152792470.pdf; https://www.thelancet.com/peer-review |
| 23 | Science immunology | American Association for the Advancement of Science | 11.19 | Northern America | Speciality areas | https://www.science.org/content/page/science-journals-editorial-policies |
| 24 | Nature Immunology | Nature Publishing Group | 10.92 | Western Europe | Speciality areas | https://www.nature.com/ni/submission-guidelines/preparing-your-submission; https://www.nature.com/nature-portfolio/editorial-policies/ai; https://www.nature.com/ni/editorial-policies/peer-review#ai-use-by-peer-reviewers |
| 25 | The Lancet Public Health | Elsevier | 10.59 | Western Europe | Speciality areas | https://www.thelancet.com/pb/assets/raw/Lancet/authors/tlpubh-info-for-authors.pdf; https://www.thelancet.com/peer-review |
| 26 | Current Protocols in Bioinformatics | Wiley | 10.41 | Northern America | General medicine | https://authorservices.wiley.com/ethics-guidelines/index.html#22 |
| 27 | Nature Reviews Microbiology | Nature Publishing Group | 10.40 | Western Europe | Speciality areas | https://www.nature.com/nrmicro/for-authors/preparing-your-submission; https://www.nature.com/nature-portfolio/editorial-policies/ai |
| 28 | Nature Reviews Gastroenterology and Hepatology | Nature Publishing Group | 10.18 | Western Europe | Speciality areas | https://www.nature.com/nrgastro/for-authors/preparing-your-submission; https://www.nature.com/nature-portfolio/editorial-policies/ai; https://www.nature.com/nature-portfolio/editorial-policies/ai |
| 29 | Journal of Clinical Oncology | American Society of Clinical Oncology | 10.16 | Northern America | Speciality areas | https://ascopubs.org/authors/journal-policies; https://ascopubs.org/authors/journal-policies#large-language-models-and-ai-tools |
| 30 | Annual Review of Public Health | Annual Reviews | 9.96 | Northern America | Speciality areas | https://www.annualreviews.org/page/authors/editorial-policies |
| 31 | The Lancet Neurology | Elsevier | 9.82 | Western Europe | Speciality areas | https://www.thelancet.com/publishing-excellence; https://www.thelancet.com/peer-review |
| 32 | Nature Cancer | Nature Publishing Group | 9.82 | Northern America | Speciality areas | https://www.nature.com/natcancer/submission-guidelines/preparing-your-submission; https://www.nature.com/natcancer/editorial-policies/peer-review#ai-use-by-peer-reviewers |
| 33 | Nature Reviews Methods Primers | Nature Publishing Group | 9.48 | Northern America | General medicine | https://www.nature.com/nrmp/for-authors/preparing-your-submission; https://www.nature.com/nature-portfolio/editorial-policies/peer-review#ai-use-by-peer-reviewers |
| 34 | The Lancet Microbe | Elsevier | 9.42 | Western Europe | Speciality areas | https://www.thelancet.com/publishing-excellence; https://www.thelancet.com/peer-review |
| 35 | Nature Microbiology | Nature Publishing Group | 9.19 | Western Europe | Speciality areas | https://www.nature.com/nmicrobiol/submission-guidelines/preparing-your-submission; https://www.nature.com/nmicrobiol/editorial-policies/peer-review#ai-use-by-peer-reviewers |
| 36 | Molecular Cancer | BioMed Central | 8.70 | Western Europe | Speciality areas | https://www.biomedcentral.com/getpublished/editorial-policies#artificial+intelligence+%28ai%29 |
| 37 | Annual Review of Pathology: Mechanisms of Disease | Annual Reviews | 8.63 | Northern America | Speciality areas | https://www.annualreviews.org/pb-assets/authors%20assets/authorhandbook-numbered.pdf |
| 38 | Gut | BMJ Publishing Group | 8.59 | Western Europe | Speciality areas | https://authors.bmj.com/policies/ai-use/ |
| 39 | Journal of the American College of Cardiology | American College of Cardiology | 8.34 | Northern America | Speciality areas | https://www.jaccsubmit.org/cgi-bin/main.plex?form_type=display_auth_instructions; https://www.jacc.org/author-center/reviewers-corner |
| 40 | The Lancet Diabetes and Endocrinology | Elsevier | 8.22 | Western Europe | Speciality areas | https://www.thelancet.com/pb/assets/raw/Lancet/authors/tlde-info-for-authors.pdf; https://www.thelancet.com/peer-review |
| 41 | The Lancet Psychiatry | Elsevier | 8.17 | Western Europe | Speciality areas | https://www.thelancet.com/pb/assets/raw/Lancet/authors/tlp-info-for-authors-1719492556657.pdf; https://www.thelancet.com/peer-review |
| 42 | Lancet Infectious Diseases | Elsevier | 8.14 | Western Europe | Speciality areas | https://www.thelancet.com/pb/assets/raw/Lancet/authors/tlid-info-for-authors-1717152767427.pdf; https://www.thelancet.com/peer-review |
| 43 | Nature Reviews Endocrinology | Nature Publishing Group | 8.12 | Western Europe | Speciality areas | https://www.nature.com/nrendo/for-authors/preparing-your-submission; https://www.nature.com/nature-portfolio/editorial-policies/ai |
| 44 | JAMA Oncology | American Medical Association | 8.10 | Northern America | Speciality areas | https://jamanetwork.com/journals/jamaoncology/pages/instructions-for-authors#SecUseofAIinPublicationandResearch |
| 45 | Circulation | American Heart Association | 7.80 | Northern America | Speciality areas | https://www.j-circ.or.jp/english/cj/Instructions_CJ.pdf |
| 46 | Gastroenterology | W.B. Saunders | 7.65 | Northern America | Speciality areas | https://www.sciencedirect.com/journal/gastroenterology/publish/guide-for-authors |
| 47 | Clinical Microbiology Reviews | American Society for Microbiology | 7.58 | Northern America | Speciality areas |  |
| 48 | Journal of Hepatology | Elsevier | 7.40 | Western Europe | Speciality areas | https://www.sciencedirect.com/journal/journal-of-hepatology/publish/guide-for-authors |
| 49 | JAMA Cardiology | American Medical Association | 7.37 | Northern America | Speciality areas | https://jamanetwork.com/journals/jamacardiology/pages/instructions-for-authors#SecUseofAIinPublicationandResearch; https://jamanetwork.com/journals/jamacardiology/pages/instructions-for-authors |
| 50 | The Lancet Global Health | Elsevier | 7.37 | Western Europe | General medicine | https://www.thelancet.com/publishing-excellence; https://www.thelancet.com/peer-review |
| 51 | Cancer Discovery | American Association for Cancer Research | 7.27 | Northern America | Speciality areas | https://aacrjournals.org/pages/editorial-policies#ai |
| 52 | Endocrine Reviews | The Endocrine Society | 7.21 | Northern America | Speciality areas | https://academic.oup.com/edrv/pages/Author_Guidelines |
| 53 | The Lancet Regional Health - Europe | Elsevier | 7.13 | Western Europe | Speciality areas | https://www.thelancet.com/pb-assets/Lancet/authors/tleurope-info-for-authors-1717152746730.pdf; https://www.thelancet.com/peer-review |
| 54 | Cell Systems | Elsevier | 7.11 | Northern America | Speciality areas | https://www.cell.com/cell-systems/authors https://www.elsevier.com/about/policies-and-standards/publishing-ethics#4-duties-of-authors; https://www.elsevier.com/about/policies-and-standards/publishing-ethics#3-duties-of-reviewers |
| 55 | Nature Metabolism | Nature Publishing Group | 7.05 | Western Europe | Speciality areas | https://www.nature.com/natmetab/submission-guidelines/preparing-your-submission https://www.nature.com/nature-portfolio/editorial-policies/ai; https://www.nature.com/natmetab/editorial-policies/peer-review#ai-use-by-peer-reviewers |
| 56 | European Urology | Elsevier | 6.96 | Western Europe | Speciality areas | https://www.sciencedirect.com/journal/european-urology/publish/guide-for-authors |
| 57 | Nature Reviews Cardiology | Nature Publishing Group | 6.93 | Western Europe | Speciality areas | https://www.nature.com/nrcardio/for-authors/preparing-your-submission; https://www.nature.com/nature-portfolio/editorial-policies/ai |
| 58 | Chem | Cell Press | 6.80 | Northern America | Speciality areas | https://www.cell.com/chem/authors; https://www.cell.com/reviewers |
| 59 | Annual Review of Clinical Psychology | Annual Reviews | 6.74 | Northern America | Speciality areas | https://www.annualreviews.org/page/authors/editorial-policies |
| 60 | JAMA Neurology | American Medical Association | 6.70 | Northern America | Speciality areas | https://jamanetwork.com/journals/jamaneurology/pages/instructions-for-authors#SecUseofAIinPublicationandResearch |
| 61 | JAMA - Journal of the American Medical Association | American Medical Association | 6.70 | Northern America | General medicine | https://jamanetwork.com/journals/jama/pages/instructions-for-authors#SecUseofAIinPublicationandResearch; https://jamanetwork.com/journals/jama/pages/instructions-for-authors#SecEditorialandPeerReview |
| 62 | Nature Biomedical Engineering | Nature Publishing Group | 6.63 | Northern America | General medicine | https://www.nature.com/natbiomedeng/submission-guidelines/preparing-your-submission https://www.nature.com/nature-portfolio/editorial-policies/ai; https://www.nature.com/natbiomedeng/editorial-policies/peer-review#ai-use-by-peer-reviewers |
| 63 | JAMA Psychiatry | American Medical Association | 6.58 | Northern America | Speciality areas | https://jamanetwork.com/journals/jamapsychiatry/pages/instructions-for-authors#SecUseofAIinPublicationandResearch; https://jamanetwork.com/journals/jamapsychiatry/pages/instructions-for-authors#SecEditorialandPeerReview |
| 64 | Nature Reviews Neurology | Nature Publishing Group | 6.54 | Western Europe | Speciality areas | https://www.nature.com/nrneurol/for-authors/preparing-your-submission https://www.nature.com/nature-portfolio/editorial-policies/ai; https://www.nature.com/nature-portfolio/editorial-policies/ai |
| 65 | Nature Reviews Nephrology | Nature Publishing Group | 6.49 | Western Europe | Speciality areas | https://www.nature.com/nrneph/for-authors/preparing-your-submission https://www.nature.com/nature-portfolio/editorial-policies/ai; https://www.nature.com/nature-portfolio/editorial-policies/ai |
| 66 | Annals of the Rheumatic Diseases | BMJ Publishing Group | 6.49 | Western Europe | Speciality areas | https://authors.bmj.com/policies/ai-use/; https://authors.bmj.com/policies/peer-review-terms-and-conditions/ |
| 67 | The Lancet Digital Health | Elsevier | 6.43 | Western Europe | Speciality areas | https://www.thelancet.com/pb-assets/Lancet/authors/tldh-info-for-authors-1717152743727.pdf; https://www.thelancet.com/peer-review |
| 68 | Accounts of Chemical Research | American Chemical Society | 6.38 | Northern America | General medicine | https://researcher-resources.acs.org/publish/author_guidelines?coden=achre4; https://pubs.acs.org/pb-assets/documents/policy/EthicalGuidelines-1676503020770.pdf |
| 69 | Science Translational Medicine | American Association for the Advancement of Science | 6.36 | Northern America | General medicine | https://www.science.org/content/page/science-journals-editorial-policies; https://www.science.org/content/page/stm-information-reviewers#guidelines |
| 70 | American Journal of Respiratory and Critical Care Medicine | American Thoracic Society | 6.24 | Northern America | Speciality areas | https://www.atsjournals.org/page/ajrccm/instructions |
| 71 | Journal of Experimental Medicine | Rockefeller University Press | 6.24 | Northern America | Speciality areas | https://rupress.org/jem/pages/editorial-policies#ai |
| 72 | Intensive Care Medicine | Springer | 6.23 | Western Europe | Speciality areas | https://link.springer.com/journal/134/submission-guidelines; https://www.springer.com/us/editorial-policies/artificial-intelligence--ai-/25428500 |
| 73 | Molecular Systems Biology | EMBO Press | 6.22 | Western Europe | General medicine | https://www.embopress.org/page/journal/17444292/authorguide; https://www.embopress.org/page/journal/17444292/refereeguide |
| 74 | The Lancet Gastroenterology and Hepatology | Elsevier | 6.17 | Western Europe | Speciality areas | https://www.thelancet.com/pb/assets/raw/Lancet/authors/tlgastro-info-for-authors-1718977668813.pdf; https://www.thelancet.com/peer-review |
| 75 | NCHS data brief | U.S. National Center for Health Statistics | 6.09 | Northern America | General medicine |  |
| 76 | Journal of Hematology and Oncology | BioMed Central | 6.05 | Western Europe | Speciality areas | https://www.biomedcentral.com/getpublished/editorial-policies#artificial+intelligence+%28ai%29 |
| 77 | Diabetes Care | American Diabetes Association | 6.01 | Northern America | Speciality areas | https://diabetesjournals.org/care/pages/instructions-for-authors; https://diabetesjournals.org/journals/pages/ada-journal-policies#artificial; https://diabetesjournals.org/journals/pages/reviewers |
| 78 | Journal of Thoracic Oncology | Elsevier | 5.87 | Northern America | Speciality areas | https://www.sciencedirect.com/journal/journal-of-thoracic-oncology/publish/guide-for-authors |
| 79 | European Journal of Heart Failure | Wiley | 5.60 | Northern America | Speciality areas | https://authorservices.wiley.com/ethics-guidelines/index.html |
| 80 | EMBO Journal | EMBO Press | 5.48 | Western Europe | General medicine | https://www.embopress.org/page/journal/14602075/authorguide; https://www.embopress.org/page/journal/14602075/refereeguide |
| 81 | Annual Review of Microbiology | Annual Reviews | 5.44 | Northern America | General medicine | https://www.annualreviews.org/page/authors/editorial-policies |
| 82 | Journal of the National Cancer Institute | Oxford University Press | 5.41 | Western Europe | Speciality areas | https://academic.oup.com/jnci/pages/General_Instructions#Editorial_Policies |
| 83 | JACC: Heart Failure | Elsevier | 5.35 | Northern America | Speciality areas | https://www.jacc.org/policies/editorial-operating-policies |
| 84 | Annual Review of Genomics and Human Genetics | Annual Reviews | 5.20 | Northern America | Speciality areas | https://www.annualreviews.org/page/authors/editorial-policies |
| 85 | Molecular Neurodegeneration | BioMed Central | 5.16 | Western Europe | Speciality areas | https://www.biomedcentral.com/getpublished/editorial-policies |
| 86 | Journal of Clinical Investigation | The American Society for Clinical Investigation | 5.12 | Northern America | General medicine | https://www.jci.org/kiosks/authors#Editorial-policies; https://www.jci.org/kiosks/ethics#Peer_review |
| 87 | JAMA Pediatrics | American Medical Association | 5.11 | Northern America | Speciality areas | https://jamanetwork.com/journals/jamapediatrics/pages/instructions-for-authors |
| 88 | Evidence-Based Mental Health | BMJ Publishing Group | 4.97 | Western Europe | Speciality areas | https://authors.bmj.com/policies/ai-use/ |
| 89 | American Journal of Human Genetics | Cell Press | 4.94 | Northern America | Speciality areas | https://www.cell.com/ajhg/authors; https://www.cell.com/reviewers |
| 90 | Progress in Retinal and Eye Research | Elsevier | 4.94 | Western Europe | Speciality areas | https://www.sciencedirect.com/journal/progress-in-retinal-and-eye-research/publish/guide-for-authors; https://www.sciencedirect.com/journal/progress-in-retinal-and-eye-research/publish/guide-for-authors |
| 91 | Blood | Elsevier | 4.93 | Northern America | Speciality areas | https://ashpublications.org/journals/pages/editorial_policies; https://ashpublications.org/blood/pages/peer-review |
| 92 | JAMA Internal Medicine | American Medical Association | 4.92 | Northern America | Speciality areas | https://jamanetwork.com/journals/jamapediatrics/pages/instructions-for-authors# |
| 93 | Genome Research | Cold Spring Harbor Laboratory Press | 4.91 | Northern America | Speciality areas | https://genome.cshlp.org/site/misc/ifora_Artificial.xhtml |
| 94 | Acta Neuropathologica | Springer | 4.90 | Western Europe | Speciality areas | https://link.springer.com/journal/401/submission-guidelines |
| 95 | Genome Medicine | BioMed Central | 4.85 | Western Europe | Speciality areas | https://www.biomedcentral.com/getpublished/editorial-policies#artificial+intelligence; https://genomebiology.biomedcentral.com/submission-guidelines/peer-review-policy |
| 96 | Neuro-Oncology | Oxford University Press | 4.83 | Western Europe | Speciality areas | https://academic.oup.com/neuro-oncology/pages/General_Instructions |
| 97 | The Lancet Haematology | Elsevier | 4.79 | Western Europe | Speciality areas | https://www.thelancet.com/publishing-excellence |
| 98 | British Journal of Sports Medicine | BMJ Publishing Group | 4.76 | Western Europe | Speciality areas | https://bjsm.bmj.com/pages/authors#editorial_policy |
| 99 | Psychotherapy and Psychosomatics | S. Karger AG | 4.74 | Western Europe | Speciality areas | https://karger.com/pages/publication-ethics#authorship; https://karger.com/pages/reviewers |
| 100 | European Respiratory Journal | European Respiratory Society | 4.72 | Western Europe | Speciality areas | https://www.ersjournals.com/authors/research-ms-preparation |
| 101 | Trends in Immunology | Cell Press | 4.69 | Western Europe | Speciality areas | https://www.cell.com/trends/immunology/authors; https://www.cell.com/reviewers |
| 102 | Clinical Cancer Research | American Association for Cancer Research Inc. | 4.60 | Northern America | Speciality areas | https://aacrjournals.org/pages/editorial-policies; https://aacrjournals.org/pages/editorial-process#peerrev |
| 103 | Lancet Rheumatology | Elsevier | 4.59 | Western Europe | Speciality areas | https://www.thelancet.com/pb-assets/Lancet/authors/tlrheum-info-for-authors-1654073741020.pdf; https://www.thelancet.com/peer-review |
| 104 | eClinicalMedicine | Elsevier | 4.55 | Western Europe | General medicine | https://www.thelancet.com/pb/assets/raw/Lancet/authors/ecm-info-for-authors-1717152732153.pdf; https://www.thelancet.com/peer-review |
| 105 | Clinical Psychology Review | Elsevier | 4.54 | Northern America | Speciality areas | https://www.sciencedirect.com/journal/clinical-psychology-review/publish/guide-for-authors; https://www.elsevier.com/about/policies-and-standards/publishing-ethics#3-duties-of-reviewers |
| 106 | Circulation Research | American Heart Association | 4.53 | Northern America | Speciality areas | https://www.ahajournals.org/submission-guidelines; https://www.ahajournals.org/for-reviewers |
| 107 | Reports on Progress in Physics | IOP Publishing | 4.47 | Western Europe | General medicine | https://publishingsupport.iopscience.iop.org/ethical-policy-journals/#generative-ai-tools |
| 108 | Journal of the American Society of Nephrology: JASN | Lippincott Williams and Wilkins | 4.45 | Northern America | Speciality areas | https://journals.lww.com/asnjournals/Pages/Information-for-Authors.aspx; https://journals.lww.com/asnjournals/pages/asnreviewerguidelines.aspx |
| 109 | Brain | Oxford University Press | 4.44 | Western Europe | Speciality areas | https://academic.oup.com/brain/pages/General_Instructions |
| 110 | JACC: Cardiovascular Imaging | Elsevier | 4.44 | Northern America | Speciality areas | https://www.jacc.org/policies/editorial-operating-policies; https://www.jacc.org/author-center/reviewers-corner#best-practices |
| 111 | Drug Resistance Updates | Churchill Livingstone | 4.39 | Northern America | Speciality areas | https://www.sciencedirect.com/journal/drug-resistance-updates/publish/guide-for-authors |
| 112 | Nature Aging | Springer Nature | 4.34 | Western Europe | Speciality areas | https://www.nature.com/nature-portfolio/editorial-policies/ai; https://www.nature.com/nataging/editorial-policies/peer-review#ai-use-by-peer-reviewers |
| 113 | eLife | eLife Sciences Publications | 4.25 | Western Europe | General medicine | https://elife-rp.msubmit.net/html/elife-rp_author_instructions.html#policies; https://elife-rp.msubmit.net/cgi-bin/main.plex?form_type=display_rev_instructions#policies |
| 114 | Annual Review of Medicine | Annual Reviews | 4.23 | Northern America | General medicine | https://www.annualreviews.org/page/authors/editorial-policies#editorial-review-process |
| 115 | Annual Review of Physical Chemistry | Annual Reviews | 4.23 | Northern America | General medicine | https://www.annualreviews.org/page/authors/editorial-policies#editorial-review-process |
| 116 | American Journal of Psychiatry | American Psychiatric Association | 4.23 | Northern America | Speciality areas | https://psychiatryonline.org/ajp/ajp_ifora |
| 117 | PLoS Medicine | Public Library of Science | 4.22 | Northern America | General medicine | https://journals.plos.org/plosmedicine/s/authorship; https://journals.plos.org/plosmedicine/s/reviewer-guidelines |
| 118 | The Lancet Child and Adolescent Health | Elsevier | 4.18 | Western Europe | Speciality areas | https://www.thelancet.com/publishing-excellence |
| 119 | Hepatology | Wolters Kluwer Health | 4.15 | Northern America | Speciality areas | https://edmgr.ovid.com/hep/accounts/ifauth.htm |
| 120 | Eurosurveillance | European Centre for Disease Prevention and Control (ECDC) | 4.15 | Western Europe | Speciality areas | https://www.eurosurveillance.org/editorial-policy#AI%20policy; https://www.eurosurveillance.org/for-reviewers |
| 121 | JAMA network open | American Medical Association | 4.11 | Northern America | General medicine | https://jamanetwork.com/journals/jamanetworkopen/pages/instructions-for-authors#SecUseofAIinPublicationandResearch; https://jamanetwork.com/journals/jamanetworkopen/pages/instructions-for-authors#SecEditorialandPeerReview |
| 122 | Journal of the National Comprehensive Cancer Network : JNCCN | Cold Spring Publishing LLC | 4.10 | Northern America | Speciality areas | https://jnccn.org/page/forauthors/information-for-authors#prep |
| 123 | Advanced Science | Wiley | 4.09 | Western Europe | General medicine | https://onlinelibrary.wiley.com/page/journal/21983844/homepage/author-guidelines https://authorservices.wiley.com/ethics-guidelines/index.html; https://authorservices.wiley.com/ethics-guidelines/index.html |
| 124 | Radiology | Radiological Society of North America | 4.07 | Northern America | Speciality areas | https://pubs.rsna.org/page/policies#llm; https://pubs.rsna.org/page/radiology/reviewers |
| 125 | Human Reproduction Update | Oxford University Press | 4.02 | Western Europe | Speciality areas | https://academic.oup.com/humupd/pages/General |
| 126 | European Journal of Epidemiology | Springer | 4.01 | Western Europe | Speciality areas | https://link.springer.com/journal/10654/submission-guidelines https://www.springer.com/us/editorial-policies/artificial-intelligence--ai-/25428500 |
| 127 | Protein Science | Wiley | 4.01 | Northern America | General medicine | https://onlinelibrary.wiley.com/page/journal/1469896x/homepage/forauthors.html |
| 128 | Clinical Infectious Diseases | Oxford University Press | 4.00 | Western Europe | Speciality areas | https://academic.oup.com/cid/pages/Policies |
| 129 | The Lancet HIV | Elsevier | 3.99 | Western Europe | Speciality areas | https://www.thelancet.com/pb/assets/raw/Lancet/authors/tlhiv-info-for-authors-1718977674203.pdf; https://www.thelancet.com/peer-review |
| 130 | Annals of Neurology | Wiley | 3.98 | Northern America | Speciality areas | https://onlinelibrary.wiley.com/page/journal/15318249/homepage/forauthors.html |
| 131 | Ophthalmology | Elsevier | 3.91 | Northern America | Speciality areas | https://www.sciencedirect.com/journal/ophthalmology/publish/guide-for-authors |
| 132 | Journal of Infection | Elsevier | 3.90 | Western Europe | Speciality areas | https://www.sciencedirect.com/journal/journal-of-infection/publish/guide-for-authors |
| 133 | Kidney International | Elsevier | 3.87 | Western Europe | Speciality areas | https://www.sciencedirect.com/journal/kidney-international/publish/guide-for-authors |
| 134 | Nano Today | Elsevier | 3.87 | Western Europe | General medicine | https://www.sciencedirect.com/journal/nano-today/publish/guide-for-authors; https://www.elsevier.com/about/policies-and-standards/publishing-ethics#3-duties-of-reviewers |
| 135 | Annals of Internal Medicine | American College of Physicians | 3.85 | Northern America | Speciality areas | https://www.acpjournals.org/pb-assets/pdf/AnnalsAuthorInfo-1719507466067.pdf |
| 136 | Arthritis and Rheumatology | Wiley | 3.79 | Western Europe | Speciality areas | https://authorservices.wiley.com/ethics-guidelines/index.html |
| 137 | Journal of Allergy and Clinical Immunology | Elsevier | 3.74 | Northern America | Speciality areas | https://www.sciencedirect.com/journal/journal-of-allergy-and-clinical-immunology/publish/guide-for-authors |
| 138 | Immunological Reviews | Wiley | 3.73 | Western Europe | Speciality areas | https://authorservices.wiley.com/ethics-guidelines/index.html |
| 139 | Microbiology and Molecular Biology Reviews | American Society for Microbiology | 3.72 | Northern America | Speciality areas | https://journals.asm.org/generative-ai; https://journals.asm.org/reviewer-guidelines |
| 140 | Microbiome | BioMed Central | 3.71 | Western Europe | Speciality areas | https://www.biomedcentral.com/getpublished/editorial-policies#artificial+intelligence+%28ai%29 |
| 141 | Blood Cancer Journal | Nature Publishing Group | 3.70 | Northern America | Speciality areas | https://www.nature.com/bjc/authors-and-referees/policies; https://www.nature.com/bcj/authors-and-referees/referees |
| 142 | GigaScience | Oxford University Press | 3.70 | Western Europe | Speciality areas | https://academic.oup.com/gigascience/pages/editorial_policies_and_reporting_standard; https://academic.oup.com/gigascience/pages/reviewer_guidelines |
| 143 | Journal of Cell Biology | Rockefeller University Press | 3.66 | Northern America | General medicine | https://rupress.org/jcb/pages/editorial-policies#ai; https://rupress.org/jcb/pages/reviewer-guidelines |
| 144 | Journal of the American Academy of Child and Adolescent Psychiatry | Elsevier | 3.66 | Northern America | Speciality areas | https://www.sciencedirect.com/journal/journal-of-the-american-academy-of-child-and-adolescent-psychiatry/publish/guide-for-authors; https://www.elsevier.com/about/policies-and-standards/publishing-ethics#4-duties-of-authors |
| 145 | JAMA Surgery | American Medical Association | 3.62 | Northern America | Speciality areas | https://jamanetwork.com/journals/jamasurgery/pages/instructions-for-authors |
| 146 | The Lancet Healthy Longevity | Elsevier | 3.62 | Western Europe | Speciality areas | https://www.thelancet.com/publishing-excellence; https://www.elsevier.com/about/policies-and-standards/publishing-ethics |
| 147 | Cancer Treatment Reviews | W.B. Saunders | 3.59 | Western Europe | Speciality areas | https://www.sciencedirect.com/journal/cancer-treatment-reviews/publish/guide-for-authors |
| 148 | Clinical Microbiology and Infection | Elsevier | 3.59 | Western Europe | Speciality areas | https://www.sciencedirect.com/journal/clinical-microbiology-and-infection/publish/guide-for-authors |
| 149 | The Lancet Planetary Health | Elsevier | 3.59 | Western Europe | Speciality areas | https://www.thelancet.com/publishing-excellence; https://www.thelancet.com/peer-review |
| 150 | Trends in Cancer | Cell Press | 3.58 | Northern America | Speciality areas | https://www.cell.com/trends/editorial-policies; https://www.cell.com/reviewers |
| 151 | Critical Care | BioMed Central | 3.58 | Western Europe | Speciality areas | https://ccforum.biomedcentral.com/submission-guidelines; https://ccforum.biomedcentral.com/submission-guidelines/peer-review-policy |
| 152 | npj Digital Medicine | Nature Publishing Group | 3.55 | Western Europe | Speciality areas | https://www.nature.com/npjdigitalmed/for-authors-and-referees/submission-guidelines; https://www.nature.com/npjdigitalmed/for-authors-and-referees/guide-to-authors |
| 153 | Cellular and Molecular Immunology | Nature Publishing Group | 3.55 | Western Europe | Speciality areas | https://www.nature.com/cmi/authors-and-referees/authors; https://www.nature.com/cmi/authors-and-referees/referees |
| 154 | Molecular Psychiatry | Nature Publishing Group | 3.54 | Western Europe | Speciality areas | https://www.nature.com/mp/authors-and-referees/editorial-policies; https://www.nature.com/mp/authors-and-referees/editorial-policies#peer-review |
| 155 | Journal of Extracellular Vesicles | Wiley | 3.53 | Northern America | Speciality areas | https://isevjournals.onlinelibrary.wiley.com/hub/journal/20013078/homepage/author-guidelines; https://authorservices.wiley.com/Reviewers/index.html |
| 156 | Health Affairs | Project HOPE | 3.50 | Northern America | Speciality areas | https://www.healthaffairs.org/help-for-authors/policies; https://www.healthaffairs.org/help-for-reviewers |
| 157 | Trends in Microbiology | Elsevier | 3.47 | Western Europe | Speciality areas | https://www.cell.com/trends/editorial-policies; https://www.cell.com/reviewers |
| 158 | Med | Cell Press | 3.46 | Northern America | General medicine | https://www.cell.com/med/authors; https://www.cell.com/reviewers |
| 159 | Nature reviews. Rheumatology | Nature Publishing Group | 3.46 | Western Europe | Speciality areas | https://www.nature.com/nrrheum/for-authors/preparing-your-submission; https://www.nature.com/nrrheum/for-referees |
| 160 | European Heart Journal | Oxford University Press | 3.45 | Western Europe | Speciality areas | https://academic.oup.com/eurheartj/pages/General_Instructions; https://academic.oup.com/eurheartj/pages/General_Instructions#peer-review-policy |
| 161 | BMC Medicine | BioMed Central | 3.45 | Western Europe | General medicine | https://www.biomedcentral.com/getpublished/editorial-policies#artificial+intelligence+%28ai%29 |
| 162 | Molecular Therapy | Cell Press | 3.43 | Northern America | General medicine | https://www.cell.com/molecular-therapy-family/molecular-therapy/authors; https://www.cell.com/reviewers |
| 163 | Journal for ImmunoTherapy of Cancer | BMJ Publishing Group | 3.40 | Western Europe | Speciality areas | https://authors.bmj.com/policies/ai-use/ |
| 164 | Small | Wiley | 3.40 | Western Europe | General medicine | https://authorservices.wiley.com/ethics-guidelines/index.html |
| 165 | Experimental and Molecular Medicine | Nature Publishing Group | 3.38 | Asiatic Region | General medicine | https://www.nature.com/documents/EMM_GTA.pdf; https://www.nature.com/emm/authors-and-referees/referees |
| 166 | Annual Review of Phytopathology | Annual Reviews | 3.38 | Northern America | General medicine | https://www.annualreviews.org/pb-assets/authors%20assets/authorhandbook-numbered.pdf |
| 167 | Leukemia | Nature Publishing Group | 3.38 | Western Europe | Speciality areas | https://www.nature.com/leu/authors-and-referees/gta; https://www.nature.com/leu/authors-and-referees/referees |
| 168 | Protein and Cell | Oxford University Press | 3.37 | Western Europe | General medicine | https://academic.oup.com/pages/authoring/journals/preparing_your_manuscript/ethics |
| 169 | American Psychologist | American Psychological Association | 3.36 | Northern America | General medicine | https://www.apa.org/pubs/journals/resources/publishing-policies |
| 170 | Journal of Clinical Epidemiology | Elsevier | 3.36 | Northern America | Speciality areas | https://www.sciencedirect.com/journal/journal-of-clinical-epidemiology/publish/guide-for-authors; https://www.elsevier.com/about/policies-and-standards/publishing-ethics#3-duties-of-reviewers |
| 171 | Diabetologia | European Association for the Study of Diabetes | 3.35 | Western Europe | Speciality areas | https://diabetologia-journal.org/for-authors-and-reviewers/instructions-to-authors/; https://diabetologia-journal.org/for-authors-and-reviewers/for-reviewers/ |
| 172 | Sports Medicine | Springer | 3.29 | Western Europe | Speciality areas | https://link.springer.com/journal/40279/submission-guidelines https://www.springer.com/us/editorial-policies/artificial-intelligence--ai-/25428500 |
| 173 | Alzheimer's and Dementia | Wiley | 3.29 | Northern America | Speciality areas | https://authorservices.wiley.com/ethics-guidelines/index.html |
| 174 | JCI insight | The American Society for Clinical Investigation | 3.28 | Northern America | General medicine | https://insight.jci.org/kiosks/submission-first#Manuscript-prep |
| 175 | Engineered Regeneration | KeAi Communications | 3.25 | Asiatic Region | General medicine | https://www.keaipublishing.com/en/journals/engineered-regeneration/guide-for-authors |
| 176 | FEMS Microbiology Reviews | Oxford University Press | 3.25 | Western Europe | Speciality areas | https://academic.oup.com/femsre/pages/Manuscript_Preparation |
| 177 | Molecular Aspects of Medicine | Elsevier | 3.24 | Western Europe | General medicine | https://www.sciencedirect.com/journal/molecular-aspects-of-medicine/publish/guide-for-authors |
| 178 | Circulation: Heart Failure | Lippincott Williams and Wilkins | 3.22 | Northern America | Speciality areas | https://www.ahajournals.org/submission-guidelines; https://www.ahajournals.org/for-reviewers |
| 179 | npj Vaccines | Nature Publishing Group | 3.20 | Western Europe | Speciality areas | https://www.nature.com/nature-portfolio/editorial-policies/ai |
| 180 | Medical Image Analysis | Elsevier | 3.20 | Western Europe | Speciality areas | https://www.sciencedirect.com/journal/medical-image-analysis/publish/guide-for-authors; https://www.elsevier.com/about/policies-and-standards/publishing-ethics#3-duties-of-reviewers |
| 181 | Journal of Neurology. Neurosurgery and Psychiatry | BMJ Publishing Group | 3.18 | Western Europe | Speciality areas | https://jnnp.bmj.com/pages/authors#editorial_policy; https://jnnp.bmj.com/pages/authors |
| 182 | The Lancet Regional Health - Americas. | Elsevier | 3.17 | Western Europe | Speciality areas | https://www.thelancet.com/publishing-excellence; https://www.thelancet.com/peer-review |
| 183 | Thorax | BMJ Publishing Group | 3.13 | Western Europe | Speciality areas | https://thorax.bmj.com/pages/authors#editorial_policy |
| 184 | Environmental Science and Technology | American Chemical Society | 3.12 | Northern America | Speciality areas | https://researcher-resources.acs.org/publish/author_guidelines?coden=esthag |
| 185 | Health Psychology Review | Routledge | 3.10 | Western Europe | Speciality areas | https://authorservices.taylorandfrancis.com/editorial-policies/defining-authorship-research-paper/; https://editorresources.taylorandfrancis.com/reviewer-guidelines/#reviewer_ethical_guidelines |
| 186 | Journal of Thrombosis and Haemostasis | Elsevier | 3.09 | Western Europe | Speciality areas | https://www.sciencedirect.com/journal/journal-of-thrombosis-and-haemostasis/publish/guide-for-authors |
| 187 | Blood advances | American Society of Hematology | 3.07 | Northern America | Speciality areas | https://ashpublications.org/journals/pages/editorial_policies#ai-policy; https://ashpublications.org/bloodadvances/pages/peer-review |
| 188 | American Journal of Obstetrics and Gynecology | Elsevier | 3.07 | Northern America | Speciality areas | https://www.sciencedirect.com/journal/american-journal-of-obstetrics-and-gynecology/publish/guide-for-authors; https://www.elsevier.com/about/policies-and-standards/publishing-ethics#3-duties-of-reviewers |
| 189 | Pharmacology and Therapeutics | Elsevier | 3.06 | Northern America | Speciality areas | https://www.sciencedirect.com/journal/pharmacology-and-therapeutics/publish/guide-for-authors; https://www.elsevier.com/about/policies-and-standards/publishing-ethics#3-duties-of-reviewers |
| 190 | Autism in Adulthood | Mary Ann Liebert | 3.06 | Northern America | Speciality areas | https://home.liebertpub.com/publications/autism-in-adulthood/646/for-authors#collapse1890; https://home.liebertpub.com/publications/autism-in-adulthood/646/for-authors#collapse1890 |
| 191 | Sleep Medicine Reviews | W.B. Saunders | 3.05 | Western Europe | Speciality areas | https://www.sciencedirect.com/journal/sleep-medicine-reviews/publish/guide-for-authors; https://www.ariessys.com/wp-content/uploads/EM-Reviewer-English.pdf |
| 192 | Emerging Infectious Diseases | Centers for Disease Control and Prevention (CDC) | 3.05 | Northern America | Speciality areas | https://wwwnc.cdc.gov/eid/page/authors-resource-instructions; https://wwwnc.cdc.gov/eid/reviewers |
| 193 | Journal of Child Psychology and Psychiatry and Allied Disciplines | Wiley | 3.03 | Western Europe | Speciality areas | https://acamh.onlinelibrary.wiley.com/hub/journal/14697610/forauthors.html |
| 194 | Journal of Internal Medicine | Wiley | 2.99 | Western Europe | Speciality areas | https://onlinelibrary.wiley.com/page/journal/13652796/homepage/forauthors.html; https://onlinelibrary.wiley.com/page/journal/13652796/homepage/guidelines-for-reviewers.htm |
| 195 | Circulation: Arrhythmia and Electrophysiology | Lippincott Williams and Wilkins | 2.99 | Northern America | Speciality areas | https://www.ahajournals.org/pb-assets/policies/EthicalConductPolicy-1661439273563.pdf; https://www.ahajournals.org/for-reviewers |
| 196 | Cancer Research | American Association for Cancer Research | 2.98 | Northern America | Speciality areas | https://aacrjournals.org/pages/editorial-policies; https://aacrjournals.org/pages/editorial-process#peerreview |
| 197 | American Journal of Kidney Diseases | W.B. Saunders | 2.97 | Northern America | Speciality areas | https://legacyfileshare.elsevier.com/promis_misc/YAJKD_InformationforAuthorsandJournalPolicies.pdf; https://sites.google.com/site/ajkdinfoforauthors/#h.p_WOC75xQ_B-M6 |
| 198 | Circulation. Genomic and precision medicine | Lippincott Williams and Wilkins | 2.97 | Northern America | Speciality areas | https://www.ahajournals.org/pb-assets/policies/EthicalConductPolicy-1661439273563.pdf; https://www.ahajournals.org/for-reviewers |
| 199 | Liver Cancer | S. Karger AG | 2.96 | Western Europe | Speciality areas | https://karger.com/lic/pages/guidelines#sec50 |
| 200 | Annals of Surgery | Lippincott Williams and Wilkins | 2.95 | Northern America | Speciality areas | https://journals.lww.com/annalsofsurgery/Pages/instructions-for-authors.aspx#Artificial%20Intelligence%20%28AI%29%20Authoring%20Tools |

**Supplementary Table 3. Journal characteristics and information sources of GAI usage guidelines of the random sample of 140 non-top SJR ranked journals**

| **ID** | **Journal name** | **Publisher** | **SJR score** | **Region** | **Focus area** | **Website** |
| --- | --- | --- | --- | --- | --- | --- |
| 1 | Alimentary Pharmacology and Therapeutics | American Academy of Family Physicians | 2.79 | Western Europe | Speciality areas | https://onlinelibrary.wiley.com/page/journal/13652036/homepage/forauthors.html?utm_source=google&utm_medium=paidsearch&utm_campaign=R3MR425&utm_content=Medicine&gad_source=1&gclid=Cj0KCQjwwO20BhCJARIsAAnTIVTpdIBbzAPqMZPzevHERwo1F-ihINrjqa-j7dwXDnBVz-kW__o4Ze8aAiRMEALw_wcB;  https://authorservices.wiley.com/ethics-guidelines/index.html |
| 2 | Journal of Clinical Hepatology | American Association for Laboratory Animal Science | 0.12 | Eastern Europe | Speciality areas | https://www.lcgdbzz.org/news/instructionsforauthors.htm;  https://www.lcgdbzz.org/news/prp.htm |
| 3 | JNCI Cancer Spectrum | American Chemical Society | 2.38 | Northern America | Speciality areas | https://academic.oup.com/jncics/pages/General_Instructions;  https://academic.oup.com/jncics/pages/General_Instructions |
| 4 | Journal of Neurosurgical Sciences | American Dental Hygienists Association | 0.49 | Western Europe | Speciality areas | https://www.minervamedica.it/en/journals/neurosurgical-sciences/notice-to-authors.php;  https://www.minervamedica.it/en/journals/neurosurgical-sciences/notice-to-authors.php |
| 5 | Obesity | American Institute of Mathematical Sciences | 1.83 | Northern America | Speciality areas | https://onlinelibrary.wiley.com/page/journal/1930739x/homepage/forauthors.html;  https://authorservices.wiley.com/ethics-guidelines/index.html#22 |
| 6 | Reviews in Endocrine and Metabolic Disorders | American Medical Association | 1.84 | Western Europe | Speciality areas | https://link.springer.com/journal/11154/submission-guidelines; https://www.springer.com/us/editorial-policies/artificial-intelligence--ai-/25428500; |
| 7 | International Journal of Health Policy and Management | BioMed Central | 1.73 | Middle East | Speciality areas | https://www.ijhpm.com/journal/authors.note;  https://www.ijhpm.com/journal/process |
| 8 | Nanotheranostics | Editorial Board of Jilin University | 0.10 | Pacific Region | General medicine | https://www.ntno.org/ms/author |
| 9 | Topics in antiviral medicine | Elsevier | 1.59 | Northern America | Speciality areas | https://www.biomedcentral.com/getpublished/editorial-policies#artificial+intelligence+%28ai%29;  https://www.biomedcentral.com/getpublished/peer-review-process |
| 10 | Current Gynecologic Oncology | Ibadan Biomedical Communications Group | 1.50 | Western Europe | Speciality areas | http://www.ginekologia.pl/index.php/instructions-for-authors;  http://www.ginekologia.pl/index.php/instructions-for-authors |
| 11 | Gynecologic and Obstetric Investigation | IOS Press BV | 1.43 | Western Europe | Speciality areas | https://karger.com/pages/publication-ethics;  https://karger.com/pages/peer-review |
| 12 | Iranian Journal of Psychiatry | Ivyspring International Publishers | 0.33 | Middle East | Speciality areas | https://ijps.tums.ac.ir/public/IRJP_Author_Guidline.pdf;  https://ijps.tums.ac.ir/index.php/ijps/peer_review_policy |
| 13 | Archives of Virology | Jacobs Verlag | 1.33 | Western Europe | General medicinel | https://www.springer.com/us/editorial-policies/artificial-intelligence--ai-/25428500; |
| 14 | Emergencias | Japanese Society for Spine Surgery and Related Research | 1.31 | Western Europe | Speciality areas | https://revistaemergencias.org/en/authors-guideline/;  https://revistaemergencias.org/en/information-for-reviewers/ |
| 15 | American Family Physician | JMIR Publications | 1.23 | Northern America | Speciality areas | https://www.aafp.org/pubs/afp/authors.html;  https://www.aafp.org/pubs/afp/reviewers.html |
| 16 | Comparative Medicine | Journal of Clinical Orthodontics | 1.28 | Northern America | General medicine | https://www.aalas.org/publications/information-for-authors/cm-and-jaalas/manuscript-preparation |
| 17 | Photodermatology Photoimmunology and Photomedicine | Kare Publishing | 1.16 | Western Europe | Speciality areas | https://authorservices.wiley.com/ethics-guidelines/index.html;  https://authorservices.wiley.com/ethics-guidelines/index.html |
| 18 | World Journal of Orthopedics | Karger | 1.16 | Asiatic Region | Speciality areas | https://www.wjgnet.com/bpg/gerinfo/204 |
| 19 | Archives of Insect Biochemistry and Physiology | Kerman University of Medical Sciences | 1.11 | Western Europe | General medicine | https://authorservices.wiley.com/ethics-guidelines/index.html;  https://authorservices.wiley.com/ethics-guidelines/index.html |
| 20 | JMIR Research Protocols | Koninklijke Belgische Vereniging voor ORL Gelaat en Halschirugie | 1.12 | Northern America | General medicine | https://www.researchprotocols.org/author-information/instructions-for-authors;  https://www.researchprotocols.org/author-information/peer-review-process |
| 21 | Orthopedics | Korea Centers for Disease Control and Prevention | 1.07 | Northern America | Speciality areas | https://journals.healio.com/journal/ortho/submit-an-article#Authors;  https://www.editorialmanager.com/orthopedics/ |
| 22 | Biomedical Signal Processing and Control | Korean Society of Lipid and Atherosclerosis | 0.15 | Western Europe | Speciality areas | https://www.sciencedirect.com/journal/biomedical-signal-processing-and-control/publish/guide-for-authors;  https://www.sciencedirect.com/journal/biomedical-signal-processing-and-control/publish/guide-for-authors |
| 23 | Spine Surgery and Related Research | Landes Bioscience | 1.03 | Asiatic Region | Speciality areas | http://ssrr-journal.jp/authors/; |
| 24 | Advances in Radiation Oncology | Lippincott Williams and Wilkins | 0.13 | Northern America | Speciality areas | https://gpsych.bmj.com/pages/authors#submission_guidelines;  https://gpsych.bmj.com/pages/authors#peer_review |
| 25 | Current Molecular Medicine | Lippincott Williams and Wilkins | 1.00 | Western Europe | General medicine | https://benthamscience.com/journal/23/ifa;  https://benthamscience.com/journal/23/ifa#research-ethics-and-policies |
| 26 | Advances in Urology | Malaysian Society for Microbiology | 1.02 | Northern America | General medicine | https://onlinelibrary.wiley.com/page/journal/9204/homepage/author-guidelines;  https://onlinelibrary.wiley.com/page/journal/9204/homepage/author-guidelines |
| 27 | Communication in Biomathematical Sciences | MDPI AG | 0.98 | Asiatic Region | Speciality areas | https://journals.itb.ac.id/index.php/cbms/ethic;  https://journals.itb.ac.id/index.php/cbms/editorialPolicies#peerReviewProcess |
| 28 | Acta Pharmaceutica | Media Sphera Publishing Group | 0.95 | Eastern Europe | General medicine | https://acta.pharmaceutica.farmaceut.org/instructions-to-authors/;  https://acta.pharmaceutica.farmaceut.org/information-to-reviewers/ |
| 29 | Pleura and Peritoneum | Medical Communications | 0.94 | Western Europe | Speciality areas | https://www.degruyter.com/publishing/for-authors/for-journal-authors/publishing-ethics;  https://www.degruyter.com/publishing/for-authors/for-journal-authors/publishing-ethics |
| 30 | Innovative Surgical Sciences | Microbiology Society | 0.93 | Western Europe | Speciality areas | https://www.degruyter.com/publishing/for-authors/for-journal-authors/publishing-ethics;  https://www.degruyter.com/publishing/for-authors/for-journal-authors/publishing-ethics |
| 31 | Journal of Pediatric Pharmacology and Therapeutics | Mosby | 0.91 | Northern America | Speciality areas | https://meridian.allenpress.com/DocumentLibrary/PPAG/JPPT_Instruction_for_Authors-12-3-2020.pdf;  https://meridian.allenpress.com/jppt/pages/Publishing-Ethics |
| 32 | ACS Synthetic Biology | Multidisciplinary Digital Publishing Institute | 0.89 | Northern America | General medicine | https://researcher-resources.acs.org/publish/author_guidelines?coden=asbcd6;  https://pubs.acs.org/pb-assets/documents/policy/EthicalGuidelines-1676503020770.pdf |
| 33 | Journal of Extracellular Vesicles | National Institute of Science Communication and Information Resources | 0.87 | Northern America | Speciality areas | https://authorservices.wiley.com/ethics-guidelines/index.html;  https://authorservices.wiley.com/ethics-guidelines/index.html |
| 34 | Journal of dental hygiene : JDH / American Dental Hygienists' Association | NIOC Health Organization | 0.86 | Northern America | Speciality areas | https://www.adha.org/wp-content/uploads/2024/03/JDH_Author_Guidelines_2024-03-01_FINAL.pdf;  https://www.adha.org/wp-content/uploads/2023/08/JDH_Ethics_Policy_2023-Aug-05.pdf |
| 35 | Journal of Biomechanics | Oxford University Press | 0.85 | Western Europe | Speciality areas | https://e-jla.org/index.php?body=instructions;  https://e-jla.org/index.php?body=peer-review_checklist |
| 36 | Jornal Brasileiro de Reproducao Assistida | Palgrave Macmillan | 0.85 | Latin America | Speciality areas | https://www.jbra.com.br/mensagem/pub/mensagem.php?id_mensagem=custom_sites&lingua_atual=_ing; |
| 37 | Cambridge Quarterly of Healthcare Ethics | Pediatric Pharmacology Advocacy Group | 0.81 | Western Europe | Speciality areas | https://www.cambridge.org/core/journals/cambridge-quarterly-of-healthcare-ethics/information/author-instructions/preparing-your-materials;  https://www.cambridge.org/core/journals/cambridge-quarterly-of-healthcare-ethics/information/peer-review-information/instructions-for-peer-reviewers |
| 38 | Folia Histochemica et Cytobiologica | Pleiades Publishing | 3.59 | Eastern Europe | Speciality areas | https://journals.viamedica.pl/folia_histochemica_cytobiologica/about/submissions;  https://journals.viamedica.pl/folia_histochemica_cytobiologica/about/customPage/aboutPlugin-page-587?adapted=1#aboutPlugin-page-587 |
| 39 | Frontiers in Bioscience - Scholar | Press of International Journal of Ophthalmology | 0.78 | Asiatic Region | General medicine | https://www.imrpress.com/journal/FBS/instructions  https://www.imrpress.com/resources/editorial_policies#5._authorship;  https://www.imrpress.com/resources/for_reviewers |
| 40 | Acta Cardiologica | PsychOpen | 2.56 | Western Europe | Speciality areas | https://taylorandfrancis.com/our-policies/ai-policy/?_ga=2.119383433.1884502056.1721688196-652683181.1721688196;  https://taylorandfrancis.com/our-policies/ai-policy/?_ga=2.119383433.1884502056.1721688196-652683181.1721688196 |
| 41 | Annals of Transplantation | Publishing House of the Romanian Academy | 0.21 | Northern America | Speciality areas | https://annalsoftransplantation.com/instructions; |
| 42 | Iranian Journal of Pharmaceutical Research | Publishing House OKI | 0.76 | Middle East | Speciality areas | https://brieflands.com/journals/ij-pharmaceutical-research/knowledgebase/category/tree;  https://brieflands.com/journals/ij-pharmaceutical-research/knowledgebase/category/tree |
| 43 | AIMS Public Health | Quintessence Publishing Company | 0.73 | Northern America | Speciality areas | https://www.aimspress.com/aimsph/news/solo-detail/instructionsforauthors;  https://www.aimspress.com/aimsph/news/solo-detail/peerreviewguidelines |
| 44 | JAMA Neurology | SAGE | 0.73 | Northern America | Speciality areas | https://jamanetwork.com/journals/jamaneurology/pages/instructions-for-authors#SecUseofAIinPublicationandResearch;  https://jamanetwork.com/journals/jamaneurology/pages/instructions-for-authors#SecEditorialandPeerReview |
| 45 | Journal of Medical Microbiology | SAGE | 0.72 | Western Europe | Speciality areas | https://www.tandfonline.com/action/authorSubmission?show=instructions&journalCode=kcbt20;  https://www.tandfonline.com/action/authorSubmission?show=instructions&journalCode=kcbt20#peers |
| 46 | Revista Brasileira de Ginecologia e Obstetricia | SAGE | 0.83 | Latin America | Speciality areas | https://www.scielo.br/j/rbgo/ |
| 47 | African Journal of Paediatric Surgery | SAGE | 0.40 | Asiatic Region | Speciality areas | https://journals.lww.com/AJPS/Pages/informationforauthors.aspx |
| 48 | Tropical Life Sciences Research | SAGE | 2.09 | Asiatic Region | General medicine | https://ejournal.usm.my/tlsr/libraryFiles/downloadPublic/11 |
| 49 | American Society of Clinical Oncology educational book / ASCO. American Society of Clinical Oncology. Meeting | Science and Medicine | 0.68 | Northern America | General medicine | https://ascopubs.org/authors/manuscript-preparation;  https://ascopubs.org/authors/peer-review-process |
| 50 | Acta Dermatovenerologica Alpina Panonica et Adriatica | Shaheed Beheshti University of Medical Sciences and Health Services | 0.10 | Eastern Europe | Speciality areas | https://acta-apa.mf.uni-lj.si/documents/Guidelines_for_authors_2023.pdf;  https://acta-apa.mf.uni-lj.si/submissions.php |
| 51 | Acta Orthopaedica Belgica | Shahid Beheshti University of Medical Sciences | 0.65 | Western Europe | Speciality areas | http://www.actaorthopaedica.be/for-authors/author-guidelines/;  http://www.actaorthopaedica.be/for-authors/author-guidelines/ |
| 52 | Cardiovascular Journal of Africa | Sichuan University | 0.66 | Africa | Speciality areas | https://www.cvja.co.za/# |
| 53 | Experimental and Clinical Transplantation | Slack Incorporated | 0.64 | Middle East | Speciality areas | https://www.ectrx.org/editorial-policy |
| 54 | Anales de Pediatria | Slovenian Medical Society | 0.64 | Western Europe | Speciality areas | https://analesdepediatria.org/en-guia-autores;  https://analesdepediatria.org/en-guia-autores |
| 55 | Journal of clinical orthodontics : JCO | Sociedade Brasileira de Reproducao Assistida | 0.62 | Northern America | Speciality areas | https://www.jco-online.com/ |
| 56 | Asia Pacific Journal of Health Management | Society for Neuroscience | 0.61 | Pacific Region | Speciality areas | https://journal.achsm.org.au/index.php/achsm/123;  https://journal.achsm.org.au/index.php/achsm/113 |
| 57 | Romanian Journal of Morphology and Embryology | South Dakota State Medical Association | 0.58 | Eastern Europe | Speciality areas | https://rjme.ro/ |
| 58 | Journal of Allied Health | Spanish Society of Emergency and Emergency Medicine | 0.60 | Northern America | Speciality areas | https://www.asahp.org/journal-of-allied-health;  https://www.asahp.org/journal-of-allied-health |
| 59 | Tanaffos | Springer | 0.57 | Middle East | Speciality areas | https://www.tanaffosjournal.ir/journal/authors.note;  https://www.tanaffosjournal.ir/journal/authors.note |
| 60 | Journal of Pediatrics | Springer | 0.57 | Northern America | Speciality areas | https://www.sciencedirect.com/journal/the-journal-of-pediatrics/publish/guide-for-authors |
| 61 | Doklady Biochemistry and Biophysics | Springer | 0.41 | Northern America | General medicine | https://link.springer.com/journal/10628;  https://www.springer.com/gp/editorial-policies/peer-review-policy-process |
| 62 | Journal of Military and Veterans' Health | Springer | 0.56 | Pacific Region | General medicine | https://jmvh.org/authors/;  https://jmvh.org/reviewers/ |
| 63 | Journal of Medical Regulation | Springer | 0.53 | Northern America | Speciality areas | https://meridian.allenpress.com/jmr/pages/For-Authors;  https://meridian.allenpress.com/jmr/pages/For-Authors#Peer-Review |
| 64 | Journal of Oral and Maxillofacial Surgery, Medicine, and Pathology | Springer | 0.53 | Western Europe | Speciality areas | https://www.sciencedirect.com/journal/journal-of-oral-and-maxillofacial-surgery-medicine-and-pathology/publish/guide-for-authors;  https://www.elsevier.com/about/policies-and-standards/publishing-ethics#4-duties-of-authors;  https://www.elsevier.com/about/policies-and-standards/publishing-ethics#3-duties-of-reviewers |
| 65 | Palliative Medicine in Practice | Springer | 0.53 | Eastern Europe | General medicine | https://journals.viamedica.pl/palliative_medicine_in_practice/about/submissions#authorGuidelines;;  https://journals.viamedica.pl/palliative_medicine_in_practice/about/customPage/aboutPlugin-page-161?adapted=1#aboutPlugin-page-161 |
| 66 | Trauma | Springer | 0.52 | Western Europe | Speciality areas | https://journals.sagepub.com/author-instructions/TRA  https://us.sagepub.com/en-us/nam/chatgpt-and-generative-ai;  https://us.sagepub.com/en-us/nam/chatgpt-and-generative-ai |
| 67 | Journal of Education and Health Promotion | Taylor & Francis | 1.07 | Asiatic Region | General medicine | https://journals.lww.com/JEHP/Pages/informationforauthors.aspx; |
| 68 | Medical Journal of Indonesia | Taylor & Francis | 0.50 | Asiatic Region | General medicine | https://mji.ui.ac.id/journal/index.php/mji/ifa;  https://mji.ui.ac.id/journal/index.php/mji/pubethics |
| 69 | Academic Forensic Pathology | Taylor & Francis | 0.79 | Western Europe | Speciality areas | https://journals.sagepub.com/author-instructions/AFP;  https://us.sagepub.com/en-us/nam/chatgpt-and-generative-ai;  https://us.sagepub.com/en-us/nam/chatgpt-and-generative-ai |
| 70 | B-ENT | Taylor & Francis | 0.49 | Western Europe | Speciality areas | http://www.b-ent.be/en/policies-103;  http://www.b-ent.be/en/policies-103 |
| 71 | South Eastern European Journal of Public Health | Taylor & Francis | 0.47 | Asiatic Region | Speciality areas | https://seejph.com/index.php/seejph/about/submissions#authorGuidelines;  https://www.seejph.com/index.php/seejph/about |
| 72 | Infectious Diseases Now | Taylor & Francis | 0.47 | Western Europe | Speciality areas | https://www.sciencedirect.com/journal/infectious-diseases-now;  https://www.elsevier.com/about/policies-and-standards/publishing-ethics#4-duties-of-authors;  https://www.elsevier.com/about/policies-and-standards/publishing-ethics#3-duties-of-reviewers |
| 73 | Journal of Sichuan University (Medical Science Edition) | Taylor & Francis | 0.46 | Asiatic Region | General medicine | http://www.jsu-mse.com/index.php/journal/pages/view/authorguidelines;  http://www.jsu-mse.com/index.php/journal/pages/view/peerreviewers |
| 74 | Formosan Journal of Surgery | Tehran University of Medical Sciences | 0.45 | Asiatic Region | Speciality areas | https://edmgr.ovid.com/fjs/accounts/ifauth.htm;  https://edmgr.ovid.com/fjs/accounts/ifauth.htm |
| 75 | Vaccines | The Allergy and Immunology Society of Thailand | 0.43 | Western Europe | Speciality areas | https://www.sciencedirect.com/journal/vaccine/publish/guide-for-authors;  https://www.sciencedirect.com/journal/vaccine/publish/guide-for-authors |
| 76 | South Dakota journal of medicine | Thieme Medical Publishers | 0.44 | Africa | General medicine | https://www.sdsma.org/AuthorSubmit |
| 77 | African Journal Biomedical Research | Tomsk National Research Medical Center of the Russian Academy of Sciences | 1.41 | Pacific Region | General medicine | https://www.ajol.info/index.php/ajbr/about/submissions |
| 78 | Immunology and Immunogenetics Insights | Universitatsklinikum Hamburg - Eppendorf | 3.53 | Northern America | Speciality areas | https://us.sagepub.com/en-us/nam/chatgpt-and-generative-ai;  https://us.sagepub.com/en-us/nam/chatgpt-and-generative-ai |
| 79 | Cellular Therapy and Transplantation | Universiti Sains Malaysia | 0.32 | Western Europe | Speciality areas | http://www.cttjournal.com/en/guidelines/ |
| 80 | Turkish Journal of Plastic Surgery | Vasile Goldis Western University of Arad | 0.70 | Asiatic Region | Speciality areas | https://journals.lww.com/tjps/Pages/informationforauthors.aspx |
| 81 | Asian Journal of Surgery | VDI Fachmedien GmBbH &amp; Co. | 0.38 | Asiatic Region | Speciality areas | https://www.sciencedirect.com/journal/asian-journal-of-surgery/publish/guide-for-authors |
| 82 | Siberian Journal of Oncology | Via Medica | 0.39 | Eastern Europe | Speciality areas | https://www.siboncoj.ru/jour/about/submissions;  https://www.siboncoj.ru/jour/about/editorialPolicies#custom-0 |
| 83 | Antibiotiki i Khimioterapiya | Via Medica | 0.37 | Middle East | Speciality areas | https://www.antibiotics-chemotherapy.ru/jour/about/submissions#authorGuidelines;  https://www.antibiotics-chemotherapy.ru/jour/about/editorialPolicies#custom-0 |
| 84 | Studia Universitatis Vasile Goldis Arad. Seria Stiintele Vietii | Weston Medical Publishing | 0.37 | Eastern Europe | General medicine | https://www.studiauniversitatis.ro/instructions/ |
| 85 | International Journal of Medical Toxicology and Forensic Medicine | Wiley | 0.36 | Middle East | Speciality areas | https://journals.sbmu.ac.ir/ijmtfm/author_guidelines;  https://journals.sbmu.ac.ir/ijmtfm/peer_review-process |
| 86 | Anatolian Journal of Family Medicine | Wiley | 0.35 | Middle East | Speciality areas | https://ajfamed.org/policies#archiving-policy;  https://ajfamed.org/policies#archiving-policy |
| 87 | Mediterranean Journal of Infection Microbes and Antimicrobials | Wiley | 0.34 | Asiatic Region | Speciality areas | https://mjima.org/static.php?id=4;  https://mjima.org/static.php?id=4 |
| 88 | International Eye Science | Wiley | 0.56 | Northern America | Speciality areas | http://www.ijo.cn/gjyken/site/menus/20220929162530001;  http://www.ijo.cn/gjyken/site/menus/20220929162550001 |
| 89 | Vascular Disease Management | Wiley | 6.70 | Eastern Europe | Speciality areas | https://www.hmpgloballearningnetwork.com/site/vdm/instructions-for-authors; |
| 90 | Wounds UK | Wiley | 0.67 | Western Europe | Speciality areas | https://wounds-uk.com/about-us/author-guidelines/;  https://wounds-uk.com/about-us/editorial-policies-and-ethics-and-malpractice-statement/ |
| 91 | Paediatria Croatica | Wolters Kluwer Medknow Publications | 0.30 | Eastern Europe | Speciality areas | https://www.paedcro.com/en/authors;  https://www.paedcro.com/en/authors |
| 92 | Indian Journal of Traditional Knowledge | Wolters Kluwer Medknow Publications | 0.13 | Asiatic Region | Speciality areas | https://nopr.niscair.res.in/jinfo/ijtk/IJTK_Inst%20to%20auth_New.pdf |
| 93 | International Journal of Occupational and Environmental Medicine | Wolters Kluwer Medknow Publications | 0.29 | Middle East | Speciality areas | https://www.sciencedirect.com/journal/best-practice-and-research-clinical-endocrinology-and-metabolism/publish/guide-for-authors;  https://www.sciencedirect.com/journal/best-practice-and-research-clinical-endocrinology-and-metabolism/publish/guide-for-authors |
| 94 | New Medicine | Wolters Kluwer Medknow Publications | 0.29 | Western Europe | General medicine | http://www.newmedicine.pl/guide/;  http://www.newmedicine.pl/guide/ |
| 95 | Technische Sicherheit | Wounds UK | 0.27 | Western Europe | Speciality areas | https://technikwissen.eu/for-authors/;  https://technikwissen.eu/for-authors/ |
| 96 | Medicine and Science in Sports and Exercise | American Society of Clinical Oncology | 0.22 | Northern America | Speciality areas | https://edmgr.ovid.com/msse/accounts/ifauth.htm |
| 97 | Forum of Clinical Oncology | ARSMB-KVBMG | 0.26 | Eastern Europe | Speciality areas | https://sciendo-parsed-data-feed.s3.eu-central-1.amazonaws.com/FCO/Author_Guidelines.pdf;  https://sciendo-parsed-data-feed.s3.eu-central-1.amazonaws.com/FCO/Author_Guidelines.pdf |
| 98 | Journal of Headache and Pain | Asociacion Espanola de Pediatria | 0.26 | Western Europe | Speciality areas | https://www.biomedcentral.com/getpublished/editorial-policies#artificial+intelligence+%28ai%29;  https://www.biomedcentral.com/getpublished/peer-review-process |
| 99 | General Psychiatry | Australasian College of Health Service Management | 0.27 | Western Europe | Speciality areas | https://gpsych.bmj.com/pages/authors#submission_guidelines;  https://gpsych.bmj.com/pages/authors#peer_review |
| 100 | Best Practice and Research in Clinical Endocrinology and Metabolism | Australasian Military Medicine Association | 0.24 | Western Europe | Speciality areas | https://www.sciencedirect.com/journal/best-practice-and-research-clinical-endocrinology-and-metabolism/publish/guide-for-authors;  https://www.sciencedirect.com/journal/best-practice-and-research-clinical-endocrinology-and-metabolism/publish/guide-for-authors |
| 101 | Acta Microbiologica et Immunologica Hungarica | Bailliere Tindall | 0.19 | Eastern Europe | Speciality areas | https://akjournals.com/page/authors;  https://akjournals.com/page/authors |
| 102 | International Journal of Sports Physiology and Performance | Baishideng Publishing Group | 0.23 | Northern America | Speciality areas | https://journals.humankinetics.com/view/journals/ijspp/ijspp-overview.xml?tab_body=author-guidelines;  https://journals.humankinetics.com/view/journals/ijspp/ijspp-overview.xml?tab_body=reviewers |
| 103 | eNeuro | Baskent University | 0.22 | Northern America | General medicine | https://www.sfn.org/about/professional-conduct/authors-of-scientific-communications#1.6; |
| 104 | Science and Medicine in Football | Bentham Science Publishers | 0.21 | Western Europe | Speciality areas | https://taylorandfrancis.com/our-policies/ai-policy/?_ga=2.119383433.1884502056.1721688196-652683181.1721688196;  https://taylorandfrancis.com/our-policies/ai-policy/?_ga=2.119383433.1884502056.1721688196-652683181.1721688196 |
| 105 | Journal of Endocrinology | BioScientifica | 1.04 | Western Europe | Speciality areas | https://joe.bioscientifica.com/page/ethics/ethical-policy;  https://joe.bioscientifica.com/page/ethics/ethical-policy |
| 106 | Archives of Toxicology | BMJ Publishing Group | 0.20 | Western Europe | General medicine | https://link.springer.com/journal/204/submission-guidelines; https://www.springer.com/us/editorial-policies/artificial-intelligence--ai-/25428500; |
| 107 | International Journal of Nanomedicine | Borgis Publishing House | 0.40 | Pacific Region | General medicine | https://taylorandfrancis.com/our-policies/ai-policy/?_ga=2.119383433.1884502056.1721688196-652683181.1721688196;  https://taylorandfrancis.com/our-policies/ai-policy/?_ga=2.119383433.1884502056.1721688196-652683181.1721688196 |
| 108 | Journal of Studies on Alcohol and Drugs | Brieflands | 0.19 | Northern America | Speciality areas | https://www.jsad.com/page/instructions |
| 109 | American Journal of Drug and Alcohol Abuse | Cambridge University Press | 0.19 | Northern America | Speciality areas | https://taylorandfrancis.com/our-policies/ai-policy/?_ga=2.119383433.1884502056.1721688196-652683181.1721688196;  https://taylorandfrancis.com/our-policies/ai-policy/?_ga=2.119383433.1884502056.1721688196-652683181.1721688196 |
| 110 | Pakistan Journal of Life and Social Sciences | Children’s University Hospital Zagreb | 0.18 | Asiatic Region | General medicine | https://www.pjlss.edu.pk/instructions.htm;  https://www.pjlss.edu.pk/instructions.htm |
| 111 | Journal of Lipid and Atherosclerosis | Clinical and Experimental Rheumatology S.A.S. | 0.17 | Asiatic Region | Speciality areas | https://e-jla.org/index.php?body=instructions;  https://e-jla.org/index.php?body=peer-review_checklist |
| 112 | Malaysian Journal of Microbiology | Clinics Cardive Publishing | 0.41 | Northern America | Speciality areas | https://mjm.usm.my/index.php?r=cms/entry/view&id=64&slug=Preparation-of-manuscript; |
| 113 | Cancer Biology and Therapy | Croatian Pharmaceutical Society | 0.17 | Northern America | Speciality areas | https://www.tandfonline.com/action/authorSubmission?show=instructions&journalCode=kcbt20;  https://www.tandfonline.com/action/authorSubmission?show=instructions&journalCode=kcbt20#peers |
| 114 | Journal of Huntington's disease | de Gruyter | 0.16 | Western Europe | Speciality areas | https://www.iospress.com/catalog/journals/journal-of-huntingtons-disease;  https://us.sagepub.com/en-us/nam/ethics-responsibility |
| 115 | Clinical and Experimental Rheumatology | de Gruyter | 0.15 | Western Europe | Speciality areas | https://www.clinexprheumatol.org/guidelines-authors.asp |
| 116 | International Journal of Oral Implantology | Edizioni Minerva Medica S.p.A. | 0.15 | Northern America | General medicine | https://www.quintessence-publishing.com/deu/en/journal/international-journal-of-oral-implantology#downloads;  https://www.quintessence-publishing.com/deu/en/journal/international-journal-of-oral-implantology#downloads |
| 117 | Journal of Diabetes Research | Elite Scientific Forum | 0.71 | Africa/Middle East | Speciality areas | https://authorservices.wiley.com/ethics-guidelines/index.html;  https://authorservices.wiley.com/ethics-guidelines/index.html |
| 118 | Microorganisms | Elsevier | 0.14 | Western Europe | Speciality areas | https://www.mdpi.com/ethics;  https://www.mdpi.com/reviewers |
| 119 | Archives of Environmental Contamination and Toxicology | Elsevier | 0.14 | Northern America | General medicine | https://link.springer.com/journal/244/submission-guidelines |
| 120 | Journal of Reproductive and Infant Psychology | Elsevier | 1.66 | Western Europe | Speciality areas | https://taylorandfrancis.com/our-policies/ai-policy/?_ga=2.119383433.1884502056.1721688196-652683181.1721688196;  https://taylorandfrancis.com/our-policies/ai-policy/?_ga=2.119383433.1884502056.1721688196-652683181.1721688196 |
| 121 | Osong Public Health and Research Perspectives | Elsevier | 0.13 | Asiatic Region | Speciality areas | https://www.ophrp.org/authors/authors.php;  https://www.ophrp.org/authors/authors.php |
| 122 | European Journal of Anaesthesiology | Elsevier | 0.13 | Western Europe | Speciality areas | https://edmgr.ovid.com/eja/accounts/ifauth.htm;  https://edmgr.ovid.com/eja/accounts/ifauth.htm |
| 123 | Asian Pacific Journal of Allergy and Immunology | Elsevier | 0.13 | Asiatic Region | Speciality areas | https://apjai-journal.org/wp-content/uploads/2023/11/author-guidelines-for-APJAI_Nov2023.pdf |
| 124 | Bioinspiration and Biomimetics | Elsevier | 0.13 | Western Europe | General medicine | https://edmgr.ovid.com/msse/accounts/ifauth.htm |
| 125 | Cancer Treatment Reviews | Elsevier | 0.11 | Western Europe | Speciality areas | https://www.sciencedirect.com/journal/cancer-treatment-reviews/publish/guide-for-authors;  https://www.elsevier.com/zh-cn/about/policies-and-standards/publishing-ethics#3-duties-of-reviewers |
| 126 | JACC: Clinical Electrophysiology | Elsevier | 0.51 | Northern America | Speciality areas | https://www.sciencedirect.com/journal/jacc-clinical-electrophysiology/publish/guide-for-authors;  https://www.sciencedirect.com/journal/jacc-clinical-electrophysiology/publish/guide-for-authors |
| 127 | Vestnik Otorinolaringologii | Elsevier | 0.12 | Eastern Europe | Speciality areas | https://www.mediasphera.ru/journal/vestnik-otorinolaringologii?expanded=true&tab=ethics;  https://www.mediasphera.ru/journal/vestnik-otorinolaringologii?expanded=true&tab=review |
| 128 | Clinical Psychology in Europe | Faculty of Medicine Universitas Indonesia | 0.12 | Western Europe | Speciality areas | https://cpe.psychopen.eu/index.php/cpe/for-authors-guidelines;  https://cpe.psychopen.eu/index.php/cpe/reviewer-policies |
| 129 | American Journal of Perinatology | Federation of State Medical Boards | 0.12 | Northern America | Speciality areas | https://www.thieme.com/en-us/journal-policies; |
| 130 | World Journal of Microbiology and Biotechnology | Galenos Publishing House | 0.11 | Western Europe | General medicine | https://www.springer.com/us/editorial-policies/artificial-intelligence--ai-/25428500 |
| 131 | Archives of Osteoporosis | Georg Thieme Verlag | 0.11 | Western Europe | Speciality areas | https://link.springer.com/journal/11657/submission-guidelines#Instructions%20for%20Authors; |
| 132 | Journal of Immunological Methods | Hellenic Society of Medical Oncology | 0.11 | Western Europe | Speciality areas | https://www.sciencedirect.com/journal/vaccine/publish/guide-for-authors;  https://www.sciencedirect.com/journal/vaccine/publish/guide-for-authors |
| 133 | Journal of Bodywork and Movement Therapies | Hindawi Publishing | 0.11 | Northern America | Speciality areas | https://www.sciencedirect.com/journal/journal-of-bodywork-and-movement-therapies/publish/guide-for-authors;  https://www.elsevier.com/about/policies-and-standards/publishing-ethics#4-duties-of-authors;  https://www.elsevier.com/about/policies-and-standards/publishing-ethics#3-duties-of-reviewers |
| 134 | Cancer Letters | HMP Communications | 0.11 | Western Europe | Speciality areas | https://www.sciencedirect.com/journal/cancer-letters/publish/guide-for-authors;  https://www.elsevier.com/about/policies-and-standards/publishing-ethics#3-duties-of-reviewers |
| 135 | Substance Abuse: Research and Treatment | Human Kinetics Publishers | 0.17 | Northern America | Speciality areas | https://us.sagepub.com/en-us/nam/chatgpt-and-generative-ai |
| 136 | Journal of Cancer Education | IMR Press Limited | 0.10 | Northern America | Speciality areas | https://www.iospress.com/catalog/journals/journal-of-huntingtons-disease;  https://us.sagepub.com/en-us/nam/ethics-responsibility |
| 137 | Open Access Rheumatology: Research and Reviews | Indonesian Biomathematical Society | 0.31 | Pacific Region | Speciality areas | https://taylorandfrancis.com/our-policies/ai-policy/?_ga=2.119383433.1884502056.1721688196-652683181.1721688196;  https://taylorandfrancis.com/our-policies/ai-policy/?_ga=2.119383433.1884502056.1721688196-652683181.1721688196 |
| 138 | American Journal of Psychoanalysis | International Antiviral Society | 0.76 | Western Europe | Speciality areas | https://www.palgrave.com/us/palgrave/journal-authors/ethics-policy/10052358;  https://www.palgrave.com/us/palgrave/journal-authors/ethics-policy/10052358 |
| 139 | Global Health Promotion | International Scientific Information | 0.10 | Western Europe | Speciality areas | https://us.sagepub.com/en-us/nam/chatgpt-and-generative-ai-0?_gl=1%2Azpzo8p%2A_ga%2AMjA1MDc5MDMyLjE3MTMyNzM1MzQ.%2A_ga_RK7MQ5ZZVZ%2AMTcyMTY4OTczMC4zNy4xLjE3MjE2ODk5MDYuMC4wLjA.%2A_gcl_au%2AMjA3ODcwODgzNS4xNzIxNjg5NzEy%2A_ga_60R758KFDG%2AMTcyMTY4OTcxMS41Ni4xLjE3MjE2ODk5MDYuNjAuMC4w%2A_ga_4FGPPZLKP6%2AMTcyMTY4OTczMC4zNy4xLjE3MjE2ODk5MDYuNDIuMC44NTQ3Nzk3Njg.;  https://us.sagepub.com/en-us/nam/chatgpt-and-generative-ai-0?_gl=1%2Azpzo8p%2A_ga%2AMjA1MDc5MDMyLjE3MTMyNzM1MzQ.%2A_ga_RK7MQ5ZZVZ%2AMTcyMTY4OTczMC4zNy4xLjE3MjE2ODk5MDYuMC4wLjA.%2A_gcl_au%2AMjA3ODcwODgzNS4xNzIxNjg5NzEy%2A_ga_60R758KFDG%2AMTcyMTY4OTcxMS41Ni4xLjE3MjE2ODk5MDYuNjAuMC4w%2A_ga_4FGPPZLKP6%2AMTcyMTY4OTczMC4zNy4xLjE3MjE2ODk5MDYuNDIuMC44NTQ3Nzk3Njg. |
| 140 | Future Science OA | IOP Publishing | 0.10 | Western Europe | General medicine | https://taylorandfrancis.com/our-policies/ai-policy/?_ga=2.119383433.1884502056.1721688196-652683181.1721688196;  https://taylorandfrancis.com/our-policies/ai-policy/?_ga=2.119383433.1884502056.1721688196-652683181.1721688196 |

**Supplementary Table 4. External GAI usage guidelines and their recommendations**

| **Guidelines on GAI usage for authors and/or reviewers** | |
| --- | --- |
| **Committee on Publication Ethics (COPE)** | The use of artificial intelligence (AI) tools such as ChatGPT or Large Language Models in research publications is expanding rapidly. COPE joins organizations, such as WAME and the JAMA Network among others, to state that AI tools cannot be listed as an author of a paper. AI tools cannot meet the requirements for authorship as they cannot take responsibility for the submitted work. As non-legal entities, they cannot assert the presence or absence of conflicts of interest nor manage copyright and license agreements. Authors who use AI tools in the writing of a manuscript, production of images or graphical elements of the paper, or in the collection and analysis of data, must be transparent in disclosing in the Materials and Methods (or similar section) of the paper how the AI tool was used and which tool was used. Authors are fully responsible for the content of their manuscript, even those parts produced by an AI tool, and are thus liable for any breach of publication ethics.  AI powered automation to increase processing speed, validation, quality assessment, and progression of the peer review process can be used, is acceptable, and even expected in many cases, provided the outcome does not result in a decision by the AI itself on acceptance or rejection of a manuscript. For example, if an AI tool detects a figure in a manuscript including a recognisable human face without the required consent form, the issue should be raised to the attention of the editor to make a decision on rejection, or the automation could proceed to automatically send a message requesting clarification, or the relevant documentation, from the authors. Publishers should take steps to be transparent about which of their publishing processes or workflows are automated, and where AI decisions are involved. Any AI powered automation should be clearly presented to the relevant participants of the peer review process—authors, reviewers, or editors—with clarification on how the algorithm provided the result or conclusion. |
| **International Committee of Medical Journal Editors (ICMJE)** | At submission, the journal should require authors to disclose whether they used artificial intelligence (AI)-assisted technologies (such as Large Language Models [LLMs], chatbots, or image creators) in the production of submitted work. Authors who use such technology should describe, in both the cover letter and the submitted work, how they used it. Chatbots (such as ChatGPT) should not be listed as authors because they cannot be responsible for the accuracy, integrity, and originality of the work, and these responsibilities are required for authorship (see Section II.A.1). Therefore, humans are responsible for any submitted material that included the use of AI-assisted technologies. Authors should carefully review and edit the result because AI can generate authoritative-sounding output that can be incorrect, incomplete, or biased. Authors should not list AI and AI-assisted technologies as an author or co-author, nor cite AI as an author. Authors should be able to assert that there is no plagiarism in their paper, including in text and images produced by the AI. Humans must ensure there is appropriate attribution of all quoted material, including full citations.  Peer Reviewers Manuscripts submitted to journals are privileged communications that are authors' private, confidential property, and authors may be harmed by premature disclosure of any or all of a manuscript's details. Reviewers therefore should keep manuscripts and the information they contain strictly confidential. Reviewers must not publicly discuss authors' work and must not appropriate authors' ideas before the manuscript is published. Reviewers must not retain the manuscript for their personal use and should destroy copies of manuscripts after submitting their reviews. Reviewers who seek assistance from a trainee or colleague in the performance of a review should acknowledge these individuals' contributions in the written comments submitted to the editor. Reviewers must maintain the confidentiality of the manuscript as outlined above, which may prohibit the uploading of the manuscript to software or other AI technologies where confidentiality cannot be assured. Reviewers should disclose to journals if and how AI technology is being used to facilitate their review. Reviewers should be aware that AI can generate authoritative-sounding output that can be incorrect, incomplete, or biased. Reviewers are expected to respond promptly to requests to review and to submit reviews within the time agreed. Reviewers' comments should be constructive, honest, and polite. Reviewers should declare their relationships and activities that might bias their evaluation of a manuscript and recuse themselves from the peer-review process if a conflict exists. |
| **World Association of Medical Editors (WAME)** | Chatbots cannot be authors. Journals have begun to publish articles in which chatbots such as Bard, Bing and ChatGPT have been used, with some journals listing chatbots as co-authors. The legal status of an author differs from country to country but under most jurisdictions, an author must be a legal person. Chatbots do not meet the International Committee of Medical Journal Editors (ICMJE) authorship criteria, particularly that of being able to give “final approval of the version to be published” and “to be accountable for all aspects of the work in ensuring that questions related to the accuracy or integrity of any part of the work are appropriately investigated and resolved.” (10) No AI tool can “understand” a conflict-of-interest statement, and does not have the legal standing to sign a statement. Chatbots have no affiliation independent of their developers. Since authors submitting a manuscript must ensure that all those named as authors meet the authorship criteria, chatbots cannot be included as authors.  Authors should be transparent when chatbots are used and provide information about how they were used. The extent and type of use of chatbots in journal publications should be indicated. This is consistent with the ICMJE recommendation of acknowledging writing assistance (11) and providing in the Methods detailed information about how the study was conducted and the results generated. (12) Authors submitting a paper in which a chatbot/AI was used to draft new text should note such use in the acknowledgment; all prompts used to generate new text, or to convert text or text prompts into tables or illustrations, should be specified. When an AI tool such as a chatbot is used to carry out or generate analytical work, help report results (e.g., generating tables or figures), or write computer codes, this should be stated in the body of the paper, in both the Abstract and the Methods section. In the interests of enabling scientific scrutiny, including replication and identifying falsification, the full prompt used to generate the research results, the time and date of query, and the AI tool used and its version, should be provided.  Authors are responsible for material provided by a chatbot in their paper (including the accuracy of what is presented and the absence of plagiarism) and for appropriate attribution of all sources (including original sources for material generated by the chatbot). Authors of articles written with the help of a chatbot are responsible for the material generated by the chatbot, including its accuracy. Noting that plagiarism is “the practice of taking someone else's work or ideas and passing them off as one's own” (13), not just the verbatim repetition of previously published text. It is the author’s responsibility to ensure that the content reflects the author's data and ideas and is not plagiarism, fabrication or falsification. Otherwise, it is potentially scientific misconduct to offer such material for publication, irrespective of how it was written. Similarly, authors must ensure that all quoted material is appropriately attributed, including full citations, and that the cited sources support the chatbot’s statements. Since a chatbot may be designed to omit sources that oppose viewpoints expressed in its output, it is the authors’ responsibility to find, review and include such counterviews in their articles. (Of course, such biases are also found in human authors.) Authors should identify the chatbot used and the specific prompt (query statement) used with the chatbot. They should specify what they have done to mitigate the risk of plagiarism, provide a balanced view, and ensure the accuracy of all their references.  Reviewer guidelines: Editors and peer reviewers should specify, to authors and each other, any use of chatbots in the evaluation of the manuscript and generation of reviews and correspondence. If they use chatbots in their communications with authors and each other, they should explain how they were used. Editors and reviewers are responsible for any content and citations generated by a chatbot. They should be aware that chatbots retain the prompts fed to them, including manuscript content, and supplying an author's manuscript to a chatbot breaches confidentiality of the submitted manuscript. |

**Supplementary Table 5. Examples of type of recommendations for author and reviewer guidelines**

| **Type of recommendations** | **Item definition** | **Item instruction (Yes)** | **Item instruction (No)** |
| --- | --- | --- | --- |
| **Author guidelines** | | | |
| Language editing | Whether GAI tools are permitted to be used in language editing | “Generative AI and AI-assisted technologies should only be used in the writing process to improve the readability and language of the manuscript.” (*Drug Target Insights*) | NA |
| Manuscript writing | Whether GAI tools are permitted to be used in manuscript writing | “If AI tools were used in the preparation of the manuscript (such as writing or revising), the authors should use the Acknowledgments section.” (*Physiological Reviews*) | “Where authors use generative artificial intelligence (AI) and AI-assisted technologies in the writing process, authors should only use these technologies to improve readability and language.” (*Gastroenterology*) |
| Data analysis and interpretation | Whether GAI tools are permitted to be used in data analysis and interpretation | “If LLMs or AI tools are used in the research itself (eg, data analysis), it must be disclosed in the Methods section.” (*Journal of Clinical Oncology*) | “Where authors use AI and AI-assisted technologies in the writing process, these technologies should only be used to improve readability and language of the work and not used to replace researcher tasks such as producing scientific insights, analysing and interpreting data, or drawing scientific conclusions.” (*The Lancet*) |
| Image generating | Whether GAI tools are permitted to be used in image generating | “It applies to all types of content, including original research, debate, opinion, journalism. It applies to all formats, including, without limitation, all text, audio, video and audio-visual material, abstracts, databases, tables, data, diagrams, photographs and other images or illustrative materials…We expect authors or others who are creating content to disclose and describe use of AI technologies in (a) any content which is submitted to us…” (*The Gut*) | “We do not permit the use of generative AI or AI-assisted tools to create or alter images in submitted manuscripts.” (*Immunity*) |
| Fact-checking | Whether authors are required to check and verify AI-generated content | “Applying these technologies should only be done with human oversight and control, and authors should carefully review and edit the result because AI can generate authoritative-sounding output that can be incorrect, incomplete, or biased.” (*The Lancet*) | NA |
| Usage documentation | Whether authors are required to disclose and document the use of GAI tools | “Use of an LLM should be properly documented in the Methods section (and if a Methods section is not available, in a suitable alternative part) of the manuscript.” (*Nature Medicine*) | NA |
| Authorship eligibility | Whether AI owns authorship and is permitted to be listed as an author | NA | “Large Language Models (LLMs), such as ChatGPT, do not currently satisfy our authorship criteria. Notably an attribution of authorship carries with it accountability for the work, which cannot be effectively applied to LLMs.” (*Nature Medicine*) |
|  | | | |
| **Reviewer guidelines** | | | |
| Language editing | Whether GAI tools are permitted to be used in language editing | “A GenAI tool can be used by an editor or peer reviewer to improve the quality of the written feedback in a peer review report.” (*Current Protocols in Bioinformatics*) | NA |
| Usage documentation | Whether reviewers are required to disclose and document the use of GAI tools | “If any part of the evaluation of the claims made in the manuscript was in any way supported by an AI tool, we ask peer reviewers to declare the use of such tools transparently in the peer review report.” (*npj Vaccines*) | NA |

Footnote: “Yes” or “No” was coded based on the item definitions provided.

NA, not applicable.

**Supplementary Table 6. Coverage of external GAI usage guidelines among top SJR ranked journals and random sample of non-top SJR ranked journals**

|  | **Top SJR ranked journals**  **(n = 182)^#^** | **Random sample of non-top SJR ranked journals (n = 116)^#^** | **P value^*^** |
| --- | --- | --- | --- |
| **Reference to external guidelines, n (%)** | | | |
| COPE | 140 (76.9) | 95 (79.3) | 0.63 |
| ICMJE | 165 (90.7) | 104 (86.2) | 0.23 |
| WAME | 40 (22.0) | 23 (18.1) | 0.42 |

Footnote: COPE, Committee on Publication Ethics; ICMJE, International Committee of Medical Journal Editors; WAME, World Association of Medical Editors. Raw counts, weighted proportions, and weighted P values were reported.

^#^ Journals referencing to any of the three external guidelines were included.

^*^ Differences between the two groups of journals were analyzed using Chi-Square tests.

**Supplementary Table 7. Linear regression analysis of the relationship between journal characteristics and number of recommendations for GAI usage guidelines among the 200 top SJR ranked journals and the random sample of 140 non-top SJR ranked journals separately**

|  | **Top SJR ranked journals**  **(n = 200)** | | | **Random sample of non-top SJR ranked journals**  **(n = 140)** | | |
| --- | --- | --- | --- | --- | --- | --- |
| **Journal characteristics** | **Coef.** | **95% CI** | **P value** | **Coef.** | **95% CI** | **P value** |
|  | **Number of recommendations of author guidelines** | | | | | |
| SJR score | -0.01 | (-0.07, 0.04) | 0.61 | -0.22 | (-0.82, 0.38) | 0.47 |
| Region |  |  |  |  |  |  |
| Northern America | Ref. | - | - | Ref. | - | - |
| Western Europe | -0.33 | (-0.84, 0.17) | 0.19 | -0.49 | (-1.26, 0.29) | 0.21 |
| Other regions^#^ | NA | NA | NA | -0.20 | (-1.21, 0.81) | 0.69 |
| Focus area |  |  |  |  |  |  |
| General medicine | Ref. | - | - | Ref. | - | - |
| Specialty areas | -0.25 | (-0.90, 0.41) | 0.46 | -0.05 | (-0.92, 0.82) | 0.91 |
|  | **Number of recommendations of reviewer guidelines** | | | | | |
| SJR score | 0.00 | (-0.01, 0.01) | 0.97 | 0.31 | (-0.17, 0.79) | 0.19 |
| Region |  |  |  |  |  |  |
| Northern America | Ref. | - | - | Ref. | - | - |
| Western Europe | -0.25 | (-0.56, 0.06) | 0.12 | -0.27 | (-1.00, 0.46) | 0.45 |
| Other regions^#^ | NA | NA | NA | -0.40 | (-1.22, 0.42) | 0.32 |
| Focus area |  |  |  |  |  |  |
| General medicine | Ref. | - | - | Ref. | - | - |
| Specialty areas | -0.03 | (-0.36, 0.41) | 0.90 | 0.05 | (-0.59, 0.69) | 0.87 |

Footnote: NA refers to ‘not available’ due to no data for these cells.

Weighted regression results were reported.

^#^ Other regions included Africa, Asiatic region, Eastern Europe, Latin America, Middle East, and Pacific region.

**Supplementary Table 8. Linear regression analysis of the relationship between journal characteristics and the number of recommendations of GAI usage guidelines after pooling two groups of journals (n = 340)**

| **Journal characteristics** | **Coef.** | **95% CI** | **P value** |
| --- | --- | --- | --- |
|  | **Number of recommendations of author guidelines** | | |
| SJR score | -0.01 | (-0.06, 0.03) | 0.57 |
| Region |  |  |  |
| Northern America | Ref. | - | - |
| Western Europe | -0.37 | (-0.79, 0.05) | 0.08 |
| Other regions^#^ | -0.10 | (-1.00, 0.80) | 0.82 |
| Focus area |  |  |  |
| General medicine | Ref. | - | - |
| Specialty areas | -0.18 | (-0.70, 0.34) | 0.48 |
|  | **Number of recommendations of reviewer guidelines** | | |
| SJR score | 0.00 | (-0.01, 0.01) | 0.94 |
| Region |  |  |  |
| Northern America | Ref. | - | - |
| Western Europe | -0.25 | (-0.54, 0.03) | 0.08 |
| Other regions^#^ | -0.38 | (-0.89, 0.14) | 0.15 |
| Focus area |  |  |  |
| General medicine | Ref. | - | - |
| Specialty areas | 0.04 | (-0.28, 0.35) | 0.80 |

Footnote: Weighted regression results were reported.

^#^ Other regions included Africa, Asiatic region, Eastern Europe, Latin America, Middle East, and Pacific region.

**Supplementary Table 9. Multinomial logistic regression examining the relationship between journal characteristics and the coverage of GAI usage guidelines among the random sample of 141 non-top SJR ranked journals (after adding one random journal without SJR score to Table 5)**

|  | **Non-top SJR journals (n = 141)** | | | | | | | | | |
| --- | --- | --- | --- | --- | --- | --- | --- | --- | --- | --- |
|  | **No guidelines** | **External guidelines only** | | | **Own guidelines only** | | | **Own & external guidelines** | | |
| **Journal characteristics** |  | **Coef.** | **95% CI** | **P value** | **Coef.** | **95% CI** | **P value** | **Coef.** | **95% CI** | **P value** |
|  |  | **Coverage of author guidelines** | | | | | | | | |
| SJR score | Ref. | 7.40 | (2.42, 12.39) | < 0.01 | 10.36 | (5.13, 15.59) | < 0.01 | 10.02 | (4.96, 15.10) | < 0.01 |
| Region |  |  |  |  |  |  |  |  |  |  |
| Northern America | Ref. | - | - | - | - | - | - | - | - | - |
| Western Europe | Ref. | 0.11 | (-1.46, 1.68) | 0.89 | 1.21 | (-1.38, 3.80) | 0.36 | 0.52 | (-0.87, 1.90) | 0.46 |
| Other regions^#^ | Ref. | 0.57 | (-0.74, 1.87) | 0.40 | -0.67 | (-3.64, 2.30) | 0.66 | -1.19 | (-2.56, 0.01) | 0.05 |
| Focus area |  |  |  |  |  |  |  |  |  |  |
| General medicine | Ref. | - | - | - | - | - | - | - | - | - |
| Specialty areas | Ref. | 0.01 | (-1.22, 1.23) | 0.99 | 0.27 | (-2.15, 2.69) | 0.83 | 0.30 | (-0.89, 1.50) | 0.62 |
|  |  | **Coverage of reviewer guidelines** | | | | | | | | |
| SJR score | Ref. | 8.27 | (3.37, 13.17) | < 0.01 | 9.48 | (4.40, 14.56) | < 0.01 | 9.27 | (4.32, 14.21) | < 0.01 |
| Region |  |  |  |  |  |  |  |  |  |  |
| Northern America | Ref. | - | - | - | - | - | - | - | - | - |
| Western Europe | Ref. | 0.15 | (-1.24, 1.54) | 0.83 | 1.21 | (-1.38, 3.80) | 0.36 | 1.00 | (-0.55, 2.54) | 0.21 |
| Other regions^#^ | Ref. | -0.24 | (-1.43, 0.95) | 0.70 | -0.67 | (-3.64, 2.30) | 0.66 | -0.83 | (-2.35, 0.70) | 0.29 |
| Focus area |  |  |  |  |  |  |  |  |  |  |
| General medicine | Ref. | - | - | - | - | - | - | - | - | - |
| Specialty areas | Ref. | 0.14 | (-1.01, 1.29) | 0.81 | 0.27 | (-2.15, 2.69) | 0.83 | 0.27 | (-1.10, 1.63) | 0.70 |

Footnote: Weighted regression results were reported.

^#^ Other regions included Africa, Asiatic region, Eastern Europe, Latin America, Middle East, and Pacific region.

**Supplementary Table 10. Multinomial logistic regression examining the relationship between journal characteristics and the coverage of GAI usage guidelines after pooling two groups of journals (n = 341) (after adding one random journal without SJR score to Table 6)**

|  | **No guidelines** | **External guidelines only** | | | **Own guidelines only** | | | **Own & external guidelines** | | |
| --- | --- | --- | --- | --- | --- | --- | --- | --- | --- | --- |
| **Journal characteristics** |  | **Coef.** | **95% CI** | **P value** | **Coef.** | **95% CI** | **P value** | **Coef.** | **95% CI** | **P value** |
|  |  | **Coverage of author guidelines** | | | | | | | | |
| SJR score | Ref. | 0.28 | (0.07, 0.49) | < 0.05 | 0.32 | (0.07, 0.57) | < 0.05 | 0.37 | (0.16, 0.57) | < 0.01 |
| Region |  |  |  |  |  |  |  |  |  |  |
| Northern America | Ref. | - | - | - | - | - | - | - | - | - |
| Western Europe | Ref. | -0.50 | (-1.59, 0.60) | 0.38 | 0.50 | (-0.96, 1.97) | 0.50 | 0.49 | (-0.47, 1.44) | 0.32 |
| Other regions^#^ | Ref. | -0.04 | (-1.03, 0.95) | 0.93 | -1.68 | (-3.99, 0.63) | 0.15 | -2.05 | (-3.10, -1.01) | < 0.01 |
| Focus area |  |  |  |  |  |  |  |  |  |  |
| General medicine | Ref. | - | - | - | - | - | - | - | - | - |
| Specialty areas | Ref. | 0.30 | (-0.65, 1.24) | 0.54 | 0.85 | (-0.85, 2.55) | 0.33 | 0.61 | (-0.25, 1.47) | 0.16 |
|  |  | **Coverage of reviewer guidelines** | | | | | | | | |
| SJR score | Ref. | 0.14 | (-0.01, 0.30) | 0.06 | 0.06 | (-0.24, 0.36) | 0.69 | 0.26 | (0.11, 0.42) | < 0.01 |
| Region |  |  |  |  |  |  |  |  |  |  |
| Northern America | Ref. | - | - | - | - | - | - | - | - | - |
| Western Europe | Ref. | -0.24 | (-1.12, 0.64) | 0.59 | 0.34 | (-1.36, 2.03) | 0.70 | 0.69 | (-0.20, 1.58) | 0.13 |
| Other regions^#^ | Ref. | -0.60 | (-1.51, 0.31) | 0.20 | -0.99 | (-3.39, 1.41) | 0.42 | -1.81 | (-3.00, -0.63) | < 0.01 |
| Focus area |  |  |  |  |  |  |  |  |  |  |
| General medicine | Ref. | - | - | - | - | - | - | - | - | - |
| Specialty areas | Ref. | 0.14 | (-0.54, 1.10) | 0.81 | 0.27 | (-1.20, 3.24) | 0.83 | 0.27 | (-0.15, 1.62) | 0.70 |

Footnote: Weighted regression results were reported.

^#^ Other regions included Africa, Asiatic region, Eastern Europe, Latin America, Middle East, and Pacific region.

**Supplementary Table 11. Number of recommendations across external GAI usage guidelines**

|  | **COPE** | **ICMJE** | **WAME** |  |
| --- | --- | --- | --- | --- |
| **Author guidelines** | | | | |
| Type of recommendations among guidelines permitting GAI usage | | | | |
| Language editing | 0 | 0 | 0 |  |
| Manuscript writing | 1 | 1 | 1 |  |
| Data analysis and interpretation | 1 | 0 | 1 |  |
| Image generating | 1 | 1 | 0 |  |
| Fact-checking | 0 | 1 | 1 |  |
| Usage documentation | 1 | 1 | 1 |  |
| Authorship eligibility | 1 | 1 | 1 |  |
| Total number of recommendations | 5 | 5 | 5 |  |
|  | | | | |
| **Reviewer guidelines** | | | | |
| Type of recommendations among guidelines permitting GAI usage | | | | |
| Language editing | 0 | 0 | 0 |  |
| Usage documentation | 1 | 1 | 1 |  |
| Total number of recommendations | 1 | 1 | 1 |  |

Footnote: “Yes” or “No” was coded based on the item definitions provided in the Methods section. A value of “1” was assigned for either “Yes” or “No” to each type of recommendation; and a value of “0” if the practice was not specified.


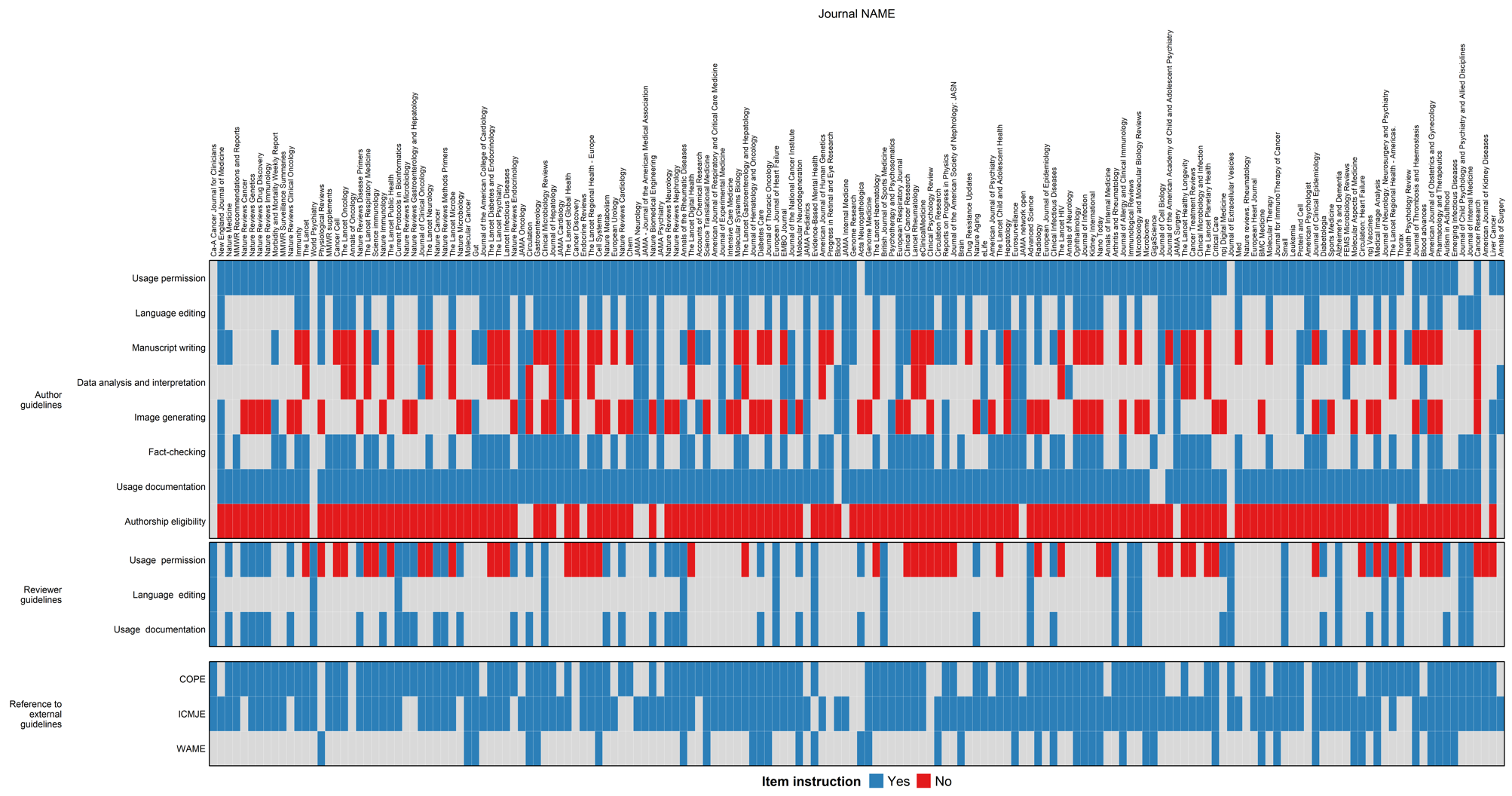
**Supplementary Figure 1. Type of recommendations across different GAI usage guidelines among top SJR ranked journals**

Footnote: “Yes” or “No” was coded based on the item definitions provided in the Methods section. The grey cells indicate that the practice was not specified.

**Supplementary Figure 2. Type of recommendations across different GAI usage guidelines among random sample of non-top SJR ranked journals**

**
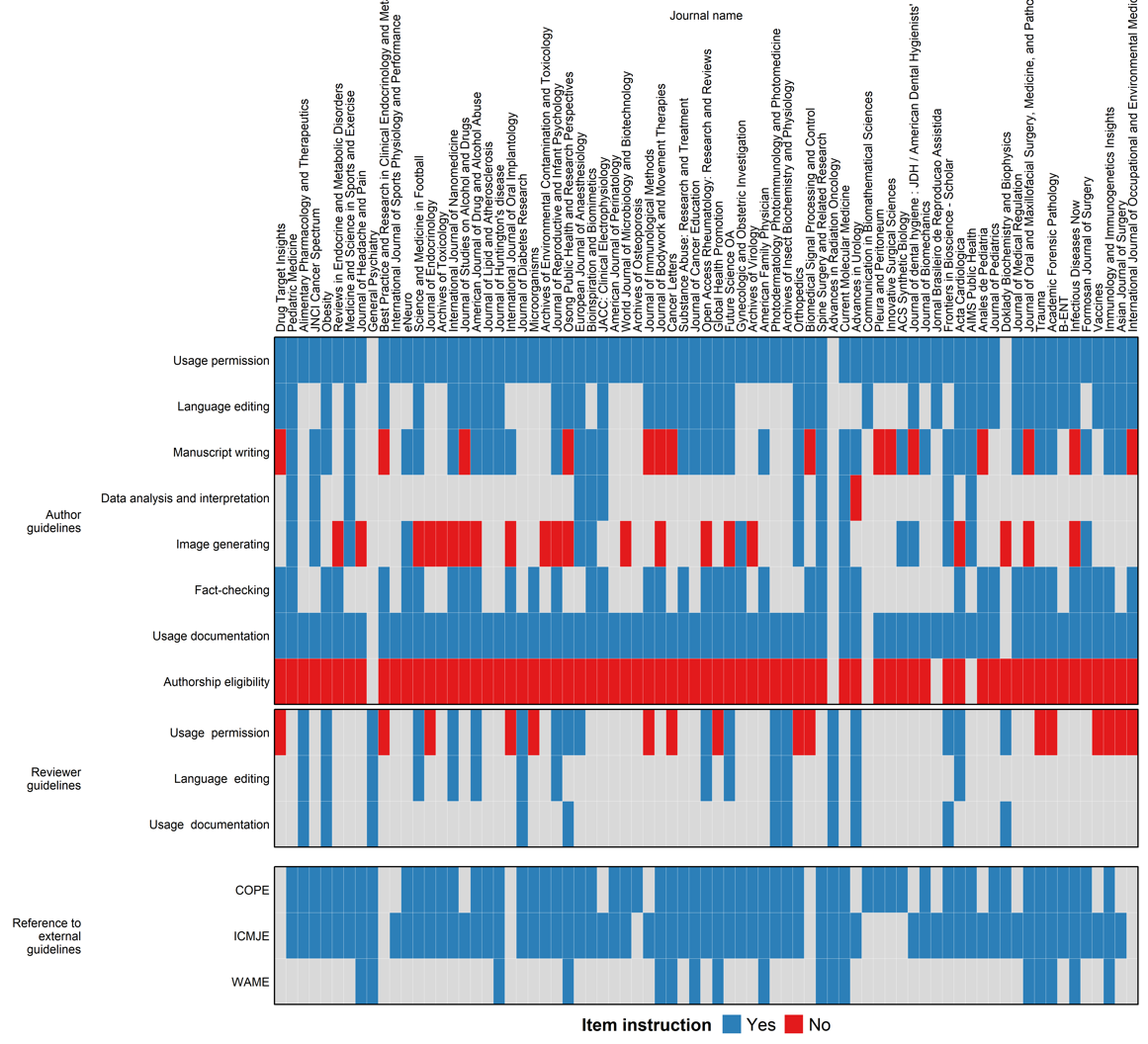
**

Footnote: “Yes” or “No” was coded based on the item definitions provided in the Methods section. The grey cells indicate that the practice was not specified.
